# Unraveling the Molecular Interactions Between Ferulic Acid and Wheat Glutenin/Gliadin in Different Systems

**DOI:** 10.3390/foods15142532

**Published:** 2026-07-17

**Authors:** Chao Chen, Meng Ding, Ruiting Li, Chongchong Wang

**Affiliations:** 1 National Key Laboratory for Development and Utilization of Forest Food Resources, Zhejiang Agriculture and Forestry University, Hangzhou 311300, China; chenchao000504@163.com (C.C.); dingmeng0521@163.com (M.D.); 15970764970@163.com (R.L.); 2 College of Food and Health, Zhejiang Agriculture and Forestry University, No. 666 Wusu Road, Linan District, Hangzhou 311300, China

**Keywords:** ferulic acid, glutenin, gliadin, interaction, aggregation behavior

## Abstract

Ferulic acid (FA) is a phenolic acid mainly present in wheat bran. It has beneficial health effects, but may affect gluten network formation and the processing quality of wheat-based products. This study investigated the interaction mechanisms between FA and glutenin/gliadin in dough and simulated dough systems. The results show that FA’s effects on both proteins were dose and system dependent. In dough, low-dose FA (≤0.3 g) promoted structural loosening of glutenin, as suggested by β-sheet conversion to β-turns/random coil structures, increased t-g-t disulfide and free thiols, and reduced particle size, whereas high doses promoted reaggregation via microenvironment reshaping, hydrophobic enhancement, cross-linking, and subunit rearrangement. For gliadin, low-dose FA may have altered local charge and hydrogen-bonding environments, while high-dose FA increased the hydrogen-bonding proportion by 45.71% and g-g-g conformation by 60.85%, suggesting enhanced molecular aggregation. In simulated dough, FA promoted stronger structural loosening of glutenin but favored gliadin aggregation, indicating that starch, lipids, water distribution, and other dough components may redirect FA–protein interactions. Molecular docking, as a complementary approach, predicted the preferential binding of FA to gliadin, LMW-GS and HMW-GS at different sites. These findings provide a theoretical basis for regulating phenolic acid–gluten interactions in whole-wheat and functional wheat-based products.

## 1. Introduction

Whole-wheat flour contains abundant dietary fiber, vitamins, minerals, and phenolic compounds, and exerts beneficial effects against chronic metabolic disorders, such as obesity, diabetes, and cardiovascular diseases. It has thus become an important direction for the global food industry’s development [1]. However, the complex non-starch polysaccharides, phenolic substances, and other components in bran, which endow whole-wheat flour products with unique health attributes, have also caused a decline in the texture and sensory quality of whole-wheat products. This situation seriously hinders industrialization and market promotion [2,3]. Dietary fiber and ferulic acid (FA), as two principal functional components in bran, exert positive effects on health. At present, the mechanism by which dietary fiber disrupts the gluten network has been largely clarified, involving reduced water availability for gluten proteins and physical interference with continuous network development [4]. Although FA accounts for only 0.5–0.7% of the total bran mass [5], its influence on flour products should not be overlooked.

FA constitutes more than 90% of the total phenolic acids in wheat bran and is its major phenolic compound [6]. The hydroxyl and phenolic acid groups present in the molecular structure of FA confer potent antioxidant properties. These properties enable FA to eliminate free radicals, inhibit lipid oxidation, and exert anti-inflammatory, anti-cancer, and metabolic regulatory effects within the body [7,8]. FA may also contribute to the maintenance of glycemic balance through enhanced insulin sensitivity and regulation of gut microbiota-derived metabolites [9]. Owing to its strong antioxidant activity, FA has been included in the list of food additives by the US Food and Drug Administration [10]. In addition, as a flavor precursor, FA is extensively utilized in the food industry.

FA has antioxidant and antimicrobial properties, which can enhance the health-promoting properties of wheat-based products [11]. FA can also alter starch digestion kinetics by hindering soluble starch digestion, inhibiting α-amylase and α-glucosidase activities, and increasing resistant starch content, thereby reducing the glycemic index of bread and meeting the increasing demand for low-GI healthy diets [12]. However, despite these functional benefits, the addition of FA may also deteriorate the quality of wheat-based products. The incorporation of FA into wheat flour dough can inhibit yeast activity, resulting in a significant reduction in bread volume. In addition, our previous studies [13,14,15] showed that FA decreased the development time and stability time of dough. The addition of FA further limited dough extensibility and fermentation capacity, leading to a denser microstructure, lower pore density, increased hardness, and reduced volume in steamed bread. However, the interactions of FA with gluten proteins and their major components, glutenin and gliadin, remain unclear.

Existing studies have suggested that FA may interact and cross-link with gluten proteins, thereby exerting either beneficial or adverse effects [16,17]. The reaction of FA with cysteine promotes the aggregation of gluten proteins, thereby affecting the formation of the gluten network [13]. Moreover, FA can further promote covalent cross-linking, thereby strengthening intermolecular interactions and facilitating the formation of a more compact and stable protein network. This structural organization is conducive to enhancing the thermal stability, mechanical properties, and water-holding ability of proteins [18,19]. However, high doses of FA may induce over-aggregation of gluten, which disrupts the integrity of the gluten network and ultimately deteriorates product quality [13]. The combined effect of FA and dietary fiber can further exacerbate quality deterioration in wheat-based products [16]. Nonetheless, the role of FA in promoting gluten protein aggregation has not been consistently confirmed. Li et al. [20] reported that FA at higher doses enhanced the polarity surrounding tryptophan residues, reduced the degree of structural organization within the gluten network, and ultimately resulted in a looser protein network.

Gluten protein, accounting for approximately 80% to 90% of the storage proteins in wheat flour and consisting mainly of glutenin and gliadin, provides the molecular basis for network formation in dough and wheat-based products [21]. Among these, glutenin possesses a comparatively higher molecular weight. The fibrous glutenin can form a continuous network framework, thereby contributing to dough strength. In contrast, gliadin is a monomeric protein with a globular structure. Owing to its poor elasticity and good fluidity, it determines the viscosity and extensibility of the dough [22,23]. Under conditions of hydration, glutenin and gliadin interact through covalent linkages as well as non-covalent interactions, forming a viscoelastic protein matrix that is closely related to the quality attributes of wheat-based products [24]. Consequently, we hypothesize that the influence of FA on gluten proteins is closely aggregated with the alterations it induces in the molecular structures of glutenin and gliadin components.

Our study provides a more in-depth investigation than previous studies. In this work, the interactions of FA with glutenin and gliadin were compared in parallel between a real dough system and a simplified simulated dough system based on isolated protein components. Unlike previous studies that mainly focused on glutenin as a whole or on FA–protein interactions in a single system, this study separately evaluated glutenin and gliadin and compared their responses under two different interaction environments. The effects of FA on the aggregation properties of glutenin and gliadin were analyzed by measuring particle size distribution and zeta potential. The changes in spatial conformation were further investigated using FT-IR spectroscopy, fluorescence spectroscopy, and Raman spectroscopy. In addition, changes in free sulfhydryl group content, chemical interaction forces, subunit composition, and SDS-PAGE patterns were determined to clarify the effects of FA on the molecular structure and cross-linking behavior of glutenin and gliadin. Finally, molecular docking was used to predict the potential binding modes between FA and glutenin/gliadin at the molecular level.

## 2. Materials and Methods

### 2.1. Materials

Wheat flour was supplied by COFCO Group (Beijing, China) Co., Ltd., and contained 12.47% protein, 12.25% moisture, 0.44% ash, and 4.73% crude fat. FA was obtained from Aladdin Reagent (Shanghai, China) Co., Ltd., with a purity of more than 99%. All other reagents used were of analytical grade or above.

### 2.2. Preparation of Glutenin and Gliadin

Gluten protein was isolated following the method described by Cao et al. [25]. In brief, wheat flour (100 g) was combined with deionized water (50 mL) and mixed at a low speed for 5 min to form dough, which was then rested in a constant temperature and humidity chamber at 30 °C for 20 min. To eliminate starch interference, the dough was rinsed with deionized water until no blue coloration appeared in the wash water after iodine testing, and wet gluten was thus obtained. The resulting gluten was stored at −80 °C for 4 h, then freeze-dried, ground, and passed through an 80-mesh sieve.

Glutenin and gliadin were isolated following the procedure reported by Wang et al. [26]. First, gluten (20 g) was mixed with dichloromethane (300 mL), stirred for 3 h, and then filtered for defatting. The defatted gluten (20 g) was then dispersed in 70% ethanol (600 mL) and stirred for another 3 h, followed by centrifugation at 4000× *g* for 20 min. The supernatant was recovered, and the ethanol was removed by rotary evaporation at 40 °C. The obtained sample was then freeze-dried, ground, and sieved through an 80-mesh screen to yield gliadin. This extraction step was carried out three times. The residue remaining after the final centrifugation was subsequently freeze-dried, ground, and passed through an 80-mesh sieve to obtain glutenin.

### 2.3. Preparation of FA-Glutenin and FA-Gliadin in Dough Systems and Simulated Dough Systems

The FA dose gradient used in this study was selected with reference to our previous studies on whole-wheat systems and wheat-based products [13]. The low FA dose was set based on the endogenous FA content in whole-wheat flour, while several higher doses were included to establish a broad concentration gradient for mechanistic investigation. Wheat flour (100 g) was thoroughly mixed with 0.1, 0.2, 0.3, 0.6, 0.9, 1.2, or 1.5 g of FA. Glutenin and gliadin were then extracted according to the method described in Section 2.2 to obtain FA-glutenin and FA-gliadin in the dough system.

Glutenin and gliadin without FA were prepared in accordance with the method described in Section 2.2. The FA-glutenin and FA-gliadin in the simulated dough system were prepared following the approach proposed by Li et al. [20]. In this study, the simulated dough system refers to a simplified protein-based model system. This model was designed to simulate the direct interaction between FA and individual gluten protein fractions during dough formation while excluding the influence of starch, lipids, and other flour components present in the actual dough system. In this system, the dough-making process was simulated by mixing isolated glutenin or gliadin with FA and water, followed by continuous kneading until a smooth, continuous, and extensible hydrated protein mass was formed. The protein mass was then rested under conditions similar to those used for dough preparation. Therefore, this system was designed to simulate the hydration, mechanical treatment, and resting environment experienced by gluten proteins during dough formation, while excluding starch, lipids, and other non-protein flour components. The simulated dough system was used to evaluate the direct interactions between FA and glutenin or between FA and gliadin under dough-like processing conditions. In addition, to maintain a protein content comparable to that in the dough system, 10 g of glutenin or gliadin was used to replace 100 g of wheat flour. Specifically, 10 g of glutenin or gliadin was thoroughly mixed with 0.1, 0.2, 0.3, 0.6, 0.9, 1.2, and 1.5 g of FA, respectively. Deionized water (10 mL) was then added, and the mixture was manually worked with a spatula until a smooth and continuous film with good extensibility was formed. The hydrated proteins were matured in a constant temperature and humidity chamber at 30 °C for 20 min, then freeze-dried, ground, and passed through an 80-mesh sieve to obtain simulated dough system FA-glutenin and FA-gliadin.

### 2.4. Particle Size Distribution

The particle size distribution was determined by referring to the method of Wang et al. [27]. The freeze-dried protein samples, which had formed specific aggregation states under different treatment conditions, were uniformly suspended in 0.5 mol/L acetic acid solution to obtain a stable dispersion system suitable for dynamic light scattering particle size measurement. Briefly, 10 mL of 0.5 mol/L acetic acid solution was slowly added to 20 mg of sample. Following shaking for 1 h, the suspension was centrifuged at 10,000× *g* for 10 min, after which the supernatant was recovered. A 2 mL portion of the supernatant was loaded into a cuvette and analyzed at 20 °C using a nanoparticle size and zeta potential analyzer (Zetasizer Nano ZS90, Malvern Instruments Ltd., Malvern, UK) in particle-size mode. All determinations were executed in triplicate, comprising 14 consecutive runs per measurement.

### 2.5. Zeta Potential

Zeta potential was measured according to a modified method based on Wang et al. [13]. Two mL of the protein solution prepared as described in Section 2.4 was introduced into a cuvette. The charge distribution of gluten protein was measured at 20 °C with a nanoparticle size and zeta potential analyzer (Zetasizer Nano ZS90, Malvern Instruments Ltd., Malvern, UK) operating in zeta mode. Each measurement was performed 14 times, and the experiment was replicated three times.

### 2.6. FTIR Spectroscopy Analysis

Protein secondary structure was characterized by FTIR spectroscopy (Nicolet 6700, Thermo Fisher Scientific Inc., Madison, WI, USA) according to a method based on Fan et al. [28]. Ten mg of sample was thoroughly mixed with 1 g of KBr and ground into a fine powder, then compressed into a thin pellet. Spectral acquisition was performed over 400–4000 cm^−1^ with 64 accumulated scans at 4 cm^−1^ resolution. PeakFit software (version 4.12, Systat Software Inc., Palo Alto, CA, USA) was applied to fit the amide I region (1600–1700 cm^−1^), from which the proportions of different secondary structural elements were calculated.

### 2.7. Fluorescence Spectroscopy Analysis

Fluorescence spectroscopy was performed with appropriate modifications based on the method described by Cao et al. [29]. A fluorescence spectrophotometer (FS5, Edinburgh Instruments Ltd., Livingston, UK) was used to determine the fluorescence spectra of the protein solution obtained in Section 2.4. The emission spectra were collected over the range of 300–400 nm with the excitation wavelength set at 290 nm.

### 2.8. Raman Spectroscopy Analysis

Raman spectroscopy was performed according to the method described by Wang et al. [30]. Raman spectra were collected using a Raman spectrometer (LabRAM Odyssey, HORIBA Scientific SAS, Palaiseau, France) equipped with a 785 nm diode laser as the excitation source. Spectra were recorded over the range of 200–1800 cm^−1^, with 800 accumulations and a spectral resolution of 1 cm^−1^. After collection, all spectra were subjected to baseline correction and normalized against the phenylalanine band at 1003 cm^−1^, which was used as an internal standard. The intensity ratio of I850/I830 was used to characterize the microenvironment of tyrosine residues, whereas I760 was used to evaluate the microenvironment of tryptophan residues. The spectral regions of 490–550 cm^−1^ were deconvoluted and processed using second-derivative analysis with PeakFit software (version 4.12, Systat Software Inc., Palo Alto, CA, USA). This region was analyzed to determine disulfide bond conformations. Peaks at 500–510 cm^−1^, 515–525 cm^−1^, and 535–545 cm^−1^ were assigned to g-g-g, t-g-g, and t-g-t conformations, respectively. The relative proportion of each disulfide bond conformation was calculated from the corresponding fitted peak area.

### 2.9. Determination of Chemical Interactions

The contributions of different chemical interaction forces were evaluated based on the method of Pan et al. [31]. The following solutions were first prepared in 0.05 mol/L phosphate buffer (pH 7.5): 0.05 mol/L NaCl (P1), 0.6 mol/L NaCl (P2), 0.6 mol/L NaCl + 1.5 M urea (P3), and 0.6 M NaCl + 8 M urea (P4). Twenty mg of sample was uniformly dispersed in 4 mL of each solution. The dispersions were stirred at 25 °C for 1 h and then centrifuged at 10,000× *g* for 20 min. A standard curve was established using bovine serum albumin, and the protein content in the supernatant was determined by the Coomassie Brilliant Blue G-250 method. The absorbance of supernatant was read at 595 nm by a multifunctional microplate reader (INFINITE E PLEX, Tecan Group Ltd., Männedorf, Switzerland).

### 2.10. Determination of Free Sulfhydryl Group Content

Free sulfhydryl group content was determined with appropriate modifications based on the method of Zhang et al. [32]. Forty mg of sample was dispersed in 4 mL of Tris-Gly buffer (0.2 mol/L, pH 8.0) containing 8 mol/L urea, 1% (*w*/*v*) SDS, and 4 mmol/L EDTA. After shaking for 1 h, the mixture was centrifuged at 10,000× *g* for 10 min, and the supernatant was collected. Then 1 mL of supernatant was reacted with 25 μL of 10 mmol/L 5,5′-dithiobis-(2-nitrobenzoic acid) (DTNB) reagent prepared in 0.2 mol/L Tris-Gly buffer. Following incubation in the dark for 20 min, absorbance at 412 nm was determined using a multifunctional microplate reader (INFINITE E PLEX, Tecan Group Ltd., Männedorf, Switzerland), with the sample solution without DTNB as the blank. Reduced glutathione was selected as the standard.

### 2.11. Reversed-Phase High-Performance Liquid Chromatography (RP-HPLC)

The subunit content within the protein was analyzed by referring to the method proposed by Hong et al. [33]. For gliadin, 50 mg of sample was dispersed in 9 mL of 60% ethanol, stirred for 1 h, and then centrifuged at 10,000× *g* for 10 min. The supernatant was collected as the gliadin solution. For glutenin, 50 mg of sample was dispersed in 3 mL of Tris-HCl buffer (0.05 mol/L, pH 7.5) containing 2.0 mol/L urea, 50% (*v*/*v*) isopropanol, and 1.0% (*w*/*v*) DTT; stirred for 1 h; and then centrifuged at 10,000× *g* for 10 min. The supernatant was recovered, and the extraction step was repeated three times. The pooled supernatants were used as the glutenin fraction. All protein extracts were passed through a 0.45 μm membrane filter, and 100 μL of each filtrate was loaded onto a Nucleosil 300-5 C8 column (Macherey-Nagel GmbH & Co. KG, Düren, Germany) connected to a Waters 1525 system (Waters Corporation, Milford, MA, USA). Deionized water and acetonitrile, each containing 0.1% (*v*/*v*) trifluoroacetic acid, served as mobile phases A and B, respectively. Separation was carried out under a linear gradient with mobile phase B increasing from 24.0% to 56.0%, at a column temperature of 50 °C, a flow rate of 1.0 mL/min, and a detection wavelength of 214 nm.

### 2.12. SDS-PAGE Analysis

SDS-PAGE analysis was performed according to the method of Abedi et al. [34]. Briefly, 5 mg of sample was dissolved in 1 mL of Tris-HCl buffer (62.5 mmol/L, pH 6.8) containing 20% glycerol, 2% SDS, 0.1% bromophenol blue, and 5% β-mercaptoethanol. For non-reducing SDS-PAGE, β-mercaptoethanol was omitted from the extraction buffer, while the remaining sample treatment procedure was the same as that used for reducing SDS-PAGE. All samples were shaken at room temperature for 4 h at 200 r/min and then centrifuged at 12,000× *g* for 15 min. The supernatants were collected, boiled for 5 min, and then cooled to room temperature. The treated supernatants (10 μL) were loaded onto gels consisting of a 12% separating gel (pH 8.8) and a 5% stacking gel (pH 6.8). Electrophoresis was initially performed at 80 V until the bromophenol blue indicator passed through the stacking gel, after which the voltage was increased to 120 V. Electrophoresis was stopped when the bromophenol blue indicator reached the bottom of the gel. The gels were carefully removed and stained for 30 min with staining solution containing 2.5 g Coomassie Brilliant Blue R-250, 45% methanol, 10% acetic acid, and 45% deionized water. The gels were then destained with destaining solution containing 7.5% methanol, 5% acetic acid, and 87.5% deionized water until the background became clear. Gel images were captured using a gel imaging system (Gel Doc XR+, Bio-Rad Laboratories Inc., Hercules, CA, USA).

### 2.13. Molecular Docking

Molecular docking was performed according to the method of Du et al. [35]. The amino acid sequences of gliadin (UniProt ID: A27319), low-molecular-weight glutenin (UniProt ID: ACK44494.1), and high-molecular-weight glutenin (UniProt ID: ACF93467.1) were obtained from the National Center for Biotechnology Information database (https://www.ncbi.nlm.nih.gov/ (accseesd on 8 September 2025)). The structure of FA was retrieved from the PubChem database (https://pubchem.ncbi.nlm.nih.gov (accseesd on 8 September 2025)). Docking between FA and the proteins was carried out using AutoDock Tools software (version 1.2.6, The Scripps Research Institute, La Jolla, CA, USA), and the interactions were visualized with Pymol (version 3.1, Schrödinger LLC, New York, NY, USA) and Discovery Studio (version 2019, Dassault Systèmes BIOVIA, San Diego, CA, USA).

### 2.14. Statistical Analysis

Each experiment was repeated three times, and the results are reported as mean ± standard deviation. Group differences were evaluated by one-way ANOVA followed by Duncan’s multiple range test (*p* < 0.05) using SPSS 27 software (SPSS Inc., Chicago, IL, USA). Figures were plotted with Origin 2024 software (OriginLab Corp., Northampton, MA, USA).

## 3. Results

### 3.1. Effects of FA on the Particle Size Distribution of Glutenin and Gliadin

**Figure 1:**
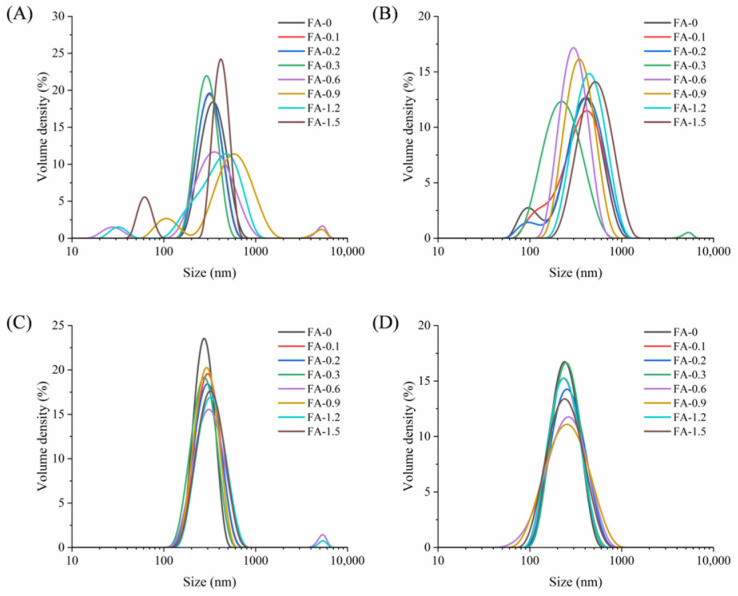
Effects of FA on the particle size distribution of glutenin and gliadin: (**A**) glutenin in the dough system; (**B**) gliadin in the dough system; (**C**) glutenin in the simulated dough system; (**D**) gliadin in the simulated dough system.

The effects of FA on the aggregation state of glutenin and gliadin in dough systems and simulated dough systems were analyzed through the determination of the particle size distribution of glutenin and gliadin. Figure 1A,B depicts the particle size distribution of glutenin and gliadin within the dough system subsequent to the addition of FA. In the dough system, the particle size distribution of glutenin in the control group was comparatively uniform. When a low dose of FA (0.3 g) was added, the peak of the particle size distribution of glutenin shifted entirely towards smaller particle sizes, and the dispersibility of protein aggregates in the acetic acid solution was improved. As the added amount of FA further increased, the main peak of glutenin shifted towards larger particle sizes, and the peak shape broadened, presenting a multi-peak distribution characteristic, suggesting an increased aggregation tendency of glutenin. The overall particle size distribution trend of gliadin in the dough system was similar to that of glutenin. When a low dose of FA was added, the gliadin particle size decreased. In contrast, a high dosage of FA led to an increase in gliadin particle size, and gliadin showed a tendency to aggregate. However, compared with glutenin, the overall change range of gliadin was smaller, indicating that glutenin was more sensitive to the effect of FA. This finding was consistent with the previous research, which indicated that FA was more likely to induce the polymerization of glutenin, and gliadin was more prone to local microenvironment remodeling [18]. In contrast, within the simulated dough system (Figure 1C,D), glutenin and gliadin exhibited a broadening of the peak shape subsequent to the addition of FA; however, the peak position did not undergo a significant change. This suggested that when FA acted directly on glutenin and gliadin, no obvious large-scale aggregation was observed. Lin et al. [19] indicated that FA was more conspicuously manifested as long-range, cross-linked network reinforcement in starch-containing reconstituted dough. By enhancing the compatibility of the starch–gluten protein interface, a more compact composite structure was formed. This suggested that the interaction between FA and gluten protein was also aggregated with component competition, network rearrangement, and processing methods during dough formation.

### 3.2. Effects of FA on Zeta Potential of Glutenin and Gliadin

**Figure 2:**
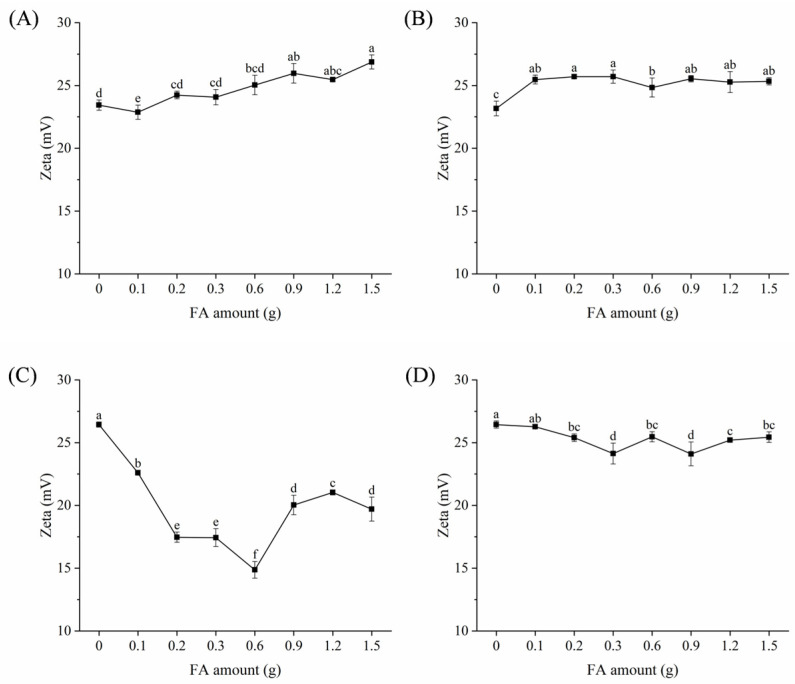
Effects of FA on the zeta potential of glutenin and gliadin: (**A**) glutenin in the dough system; (**B**) gliadin in the dough system; (**C**) glutenin in the simulated dough system; (**D**) gliadin in the simulated dough system. Different lowercase letters within a column denote significant difference at *p* < 0.05.

Zeta potential analysis provides information on the surface charge properties of proteins and is important for elucidating protein–protein interactions, particularly electrostatic repulsion [36]. The findings revealed that FA affected the surface charge behavior of glutenin and gliadin particles in a distinctly different manner across the two systems (Figure 2A–D). In the dough system, the zeta potential value of glutenin generally increased with the increase in FA, while that of gliadin rapidly rose at an FA dose of 0.1 g and then stabilized. This indicated that FA enhanced the electrostatic repulsion between protein particles, improving the dispersion stability of the system. This finding was in line with the report by Wang et al. [13], which suggested that FA could alter the spatial structure of gluten, resulting in a more extended protein structure and an increase in zeta potential. Notably, although the zeta potential of glutenin and gliadin was still significantly higher than that of the control group at high doses of FA, the particle size distribution showed a rightward shift in the distribution peak, indicating that the aggregation was unlikely to be mainly driven by charge neutralization. In the simulated dough system, the zeta potential of both glutenin and gliadin was significantly lower than that of the control group with the addition of FA, and the decrease in glutenin was more pronounced. This suggested that the direct effect of FA in the pure protein system was more inclined to weaken the stability of the surface charge of the particles. Combined with the broadening of the particle size distribution peak of glutenin and gliadin in the simulated dough system, this indicated that the system became more susceptible to slight aggregation following the reduction in electrostatic repulsion. However, in the absence of a true dough network and starch participation, this effect was not sufficient to form a large number of large-scale aggregates.

### 3.3. Effects of FA on the Secondary Structure of Glutenin and Gliadin

**Figure 3:**
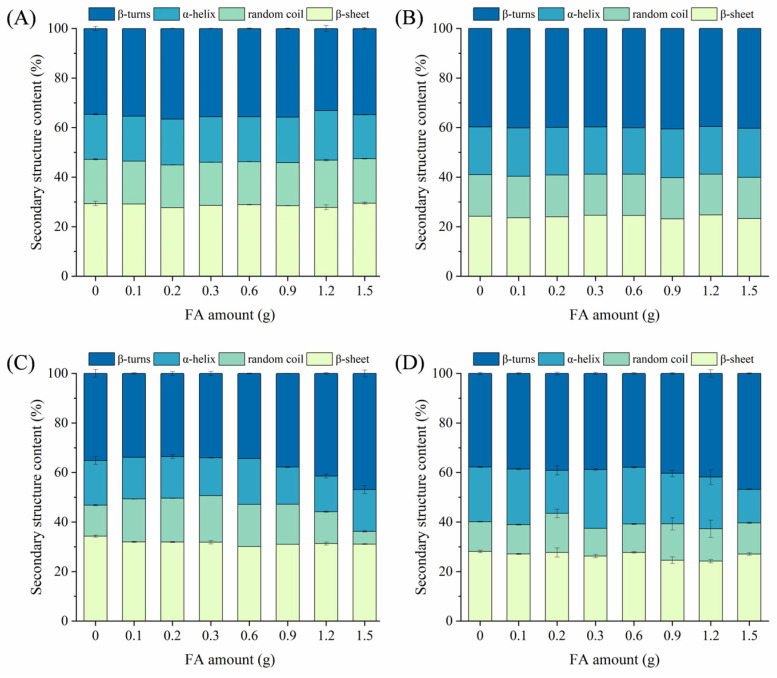
Effects of FA on the secondary structure of glutenin and gliadin: (**A**) glutenin in the dough system; (**B**) gliadin in the dough system; (**C**) glutenin in the simulated dough system; (**D**) gliadin in the simulated dough system.

The amide I band (1600–1700 cm^−1^) in FTIR spectroscopy is widely employed for the characterization of protein secondary structure [37]. Alterations in secondary-structure composition directly affect the structural strength and extensibility of gluten and consequently exert a marked influence on dough quality [38]. The effects of FA on the relative contents of secondary structures of glutenin and gliadin are shown in Figure 3A–D. The FTIR spectra and the corresponding deconvoluted amide I bands are presented in Figures S1–S5. In the dough system, the content of β-sheet in glutenin slightly decreased and the content of β-turn relatively increased after low-dose FA treatment, indicating that FA appeared to partially alter the original ordered structure of glutenin to some extent and promoted its transformation into a more extended structure. However, when the FA addition reached 1.2 g, the content of α-helix significantly increased by 10.15% compared with the control group, while the content of β-turn significantly decreased by 4.39%. This indicated that this change may be aggregated with further aggregation and possible cross-linking of glutenin molecules and facilitated the formation of a dense network structure. The secondary structure of gliadin in the dough system remained relatively stable, showing only slight fluctuations, indicating that it was less responsive to FA. In the simulated dough system, the changes in the secondary structure of glutenin were more significant. With the increase in the addition of FA, the content of β-sheet generally decreased, while the content of β-turn significantly increased at higher addition levels. When 1.5 g of FA was added, the content of β-turn increased by 33.37% compared with the control group. This indicated that when FA directly acted on glutenin, it significantly disrupted its ordered structure and enhanced the disorder of chain segments. The secondary structure changes of gliadin in the simulated dough system were consistent with those of glutenin. Huang et al. [39] reported that FA could induce different degrees of rearrangement of the secondary structure of gluten, manifested as the transformation of β-sheet to β-turn, which was consistent with the results of the simulated dough system in this study. The decrease in the β-sheet content and the increase in the β-turn content of gliadin and glutenin indicated that FA could disturb the relatively ordered structure of the original protein and induce local conformational adjustments.

### 3.4. Effects of FA on Fluorescence Spectra of Glutenin and Gliadin

**Figure 4:**
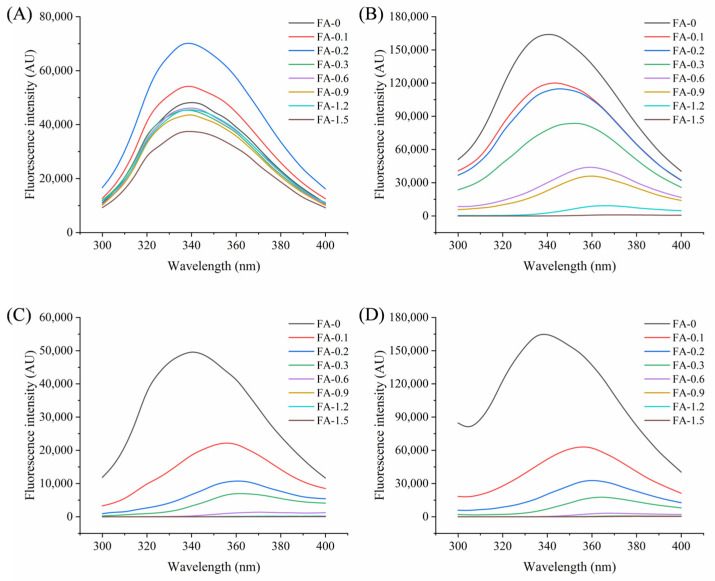
Effects of FA on the fluorescence spectra of glutenin and gliadin: (**A**) glutenin in the dough system; (**B**) gliadin in the dough system; (**C**) glutenin in the simulated dough system; (**D**) gliadin in the simulated dough system.

Protein fluorescence spectra can reveal alterations in the microenvironment surrounding the three fluorescent amino acids in gluten proteins, including tryptophan (Trp), tyrosine (Tyr), and phenylalanine (Phe). Among them, Trp has the highest fluorescence intensity, and most proteins contain Trp residues that are the most sensitive to changes in the polarity of the microenvironment. Therefore, Trp is often used as an endogenous fluorescence probe to study the conformation of proteins in solution [40,41]. Figure 4A–D illustrate the effects of FA on the fluorescence spectra of glutenin and gliadin within different systems. In the dough system, a marginal increase in the fluorescence intensity of glutenin was observed when FA was added at concentrations below 0.6 g; however, fluorescence quenching commenced when FA supplementation exceeded 0.6 g. At low doses, FA may have induced partial relaxation of glutenin aggregates in dough, increasing the fluorescence quantum yield by reducing non-radiative energy loss [42]. However, at high FA doses, the maximum peak intensity of gluten proteins began to be lower than that of the control group samples. At this point, the interaction between FA and proteins intensified, possibly forming a cross-linked complex of FA and glutenin, leading to fluorescence quenching [43]. It is worth noting that within the entire concentration range, the maximum fluorescence emission wavelength did not change significantly, indicating that the polarity of the Trp microenvironment in glutenin did not undergo substantial alteration. In contrast, the gliadin in the dough system showed significant fluorescence quenching with the addition of FA, accompanied by a distinct red shift in the maximum fluorescence emission wavelength. This indicated an increase in the polarity of the Trp microenvironment, suggesting that the addition of FA induced the unfolding of the protein conformation and suggesting possible exposure of aromatic residues to a more polar environment [44]. Lin et al. [18] proposed that the structural plasticity of gliadin enabled FA to preferentially interact with its hydrophobic domains, thereby resulting in a pronounced red shift in fluorescence emission peaks upon FA incorporation, which was consistent with the results of this study. In the simulated dough system, the fluorescence spectra of glutenin and gliadin showed a consistent trend of change. With the addition of FA, the fluorescence intensity of the protein decreased significantly, accompanied by a significant red shift in the maximum fluorescence absorption wavelength, and the change trend was more obvious than that of gliadin in the dough system. This was consistent with the research results of Li et al. [20], who found that when FA directly acted on gluten proteins gliadin and glutenin, it could enhance the polarity of the microenvironment of tryptophan residues and expose the hydrophobic groups of proteins, thereby reducing fluorescence intensity. Compared with the dough system, in the simulated dough system, there was no interference from complex non-protein components such as starch, and the interaction between FA and glutenin and gliadin was more direct, which may have resulted in stronger fluorescence quenching and obvious red shift. The dough matrix may attenuate the direct interaction between FA and glutenin and gliadin by applying steric hindrance and competitive binding effects, thereby alleviating the conformational response detected by fluorescence spectroscopy.

### 3.5. Effects of FA on Raman Spectra of Glutenin and Gliadin

#### 3.5.1. Analysis of Amino Acid Side Chains

Raman spectroscopy is utilized to characterize the microenvironment of amino acid side chains in gluten protein molecules, thereby elucidating changes in their tertiary structure. The Raman spectra are shown in Figure S6. Specifically, the characteristic vibrational frequencies generated by the ring-breathing and out-of-plane bending vibrations of Tyr are located around 830 cm^−1^ and 850 cm^−1^. The relative intensity of this doublet (I850/I830) is frequently employed to determine the degree of burial or exposure of Tyr residues [45]. The intensity ratio of the Tyr doublet is presented in Table 1. In glutenin from the dough system, the Tyr doublet ratio decreased significantly after the addition of FA. At an FA dose of 0.9 g, the Tyr doublet ratio decreased by 26.11% compared with the control. This result indicated that FA altered the local hydrogen-bonding environment around Tyr residues in glutenin, possibly causing Tyr residues to be located in a more spatially restricted or aggregated microenvironment. In gliadin from the dough system, the Tyr doublet ratio fluctuated after the addition of FA, but showed an overall increase under high FA doses. This suggested that FA changed the microenvironment of Tyr residues in gliadin, especially at high doses, which may be related to the disruption of gliadin tertiary structure by high-dose FA, leading to increased exposure of Tyr residues or rearrangement of the surrounding hydrogen-bonding network. This result was also consistent with the fluorescence spectroscopy results, indicating that the amino acid microenvironment of gliadin was more sensitive to FA in the dough system. In the simulated dough system, the Tyr doublet ratio of glutenin generally decreased after the addition of FA. This confirmed that the direct interaction between FA and isolated glutenin could alter the Tyr microenvironment and local hydrogen-bonding state. For gliadin in the simulated system, the Tyr doublet ratio showed a fluctuating pattern and increased significantly at 0.3 and 0.9 g FA. These results indicated that the effect of FA on the Tyr microenvironment depended on protein type and matrix system, rather than being determined solely by FA dose. Similar phenomena were also reported by Li et al. [20], who found that the direct interaction between FA and gluten proteins significantly changed the Tyr doublet ratio.

In Raman spectroscopy, the band near 760 cm^−1^ is assigned to the Trp side chain (Table 1). A decrease in this band’s intensity signifies the exposure of Trp residues to a hydrophilic environment, whereas an increase in intensity indicates the burial of Trp residues within a hydrophobic environment [46]. For glutenin in the dough system, the intensity of the Trp band generally increased with the addition of FA. This suggested that following FA treatment, the Trp residues progressively entered a more buried, hydrophobic, and restricted local environment, indicating that glutenin may have undergone rearrangement toward localized aggregate structures [47]. In contrast, except at the low FA dose, the Trp band intensity of gliadin in the dough system was lower than that of the control. This indicated that when FA interacted with gliadin in the dough system and with isolated glutenin and gliadin, it significantly induced the unfolding of the protein tertiary structure, leading to the exposure of Trp residues. In dough, glutenin is a linear polymer composed of high-molecular-weight (HMW) and low-molecular-weight (LMW) glutenin subunits linked by intermolecular disulfide bonds, and therefore has strong cross-linking and rearrangement potential [48]. However, in the simulated dough system, FA exerted markedly different effects on the Trp residues of glutenin and gliadin. With the addition of FA, the Trp band intensity of glutenin in the simulated dough system decreased significantly, indicating that FA disturbed the original hydrophobic microenvironment of Trp and exposed it more to a polar environment [18]. This further confirmed that FA induced structural loosening of isolated glutenin. In contrast, the Trp band intensity of gliadin in the simulated system increased significantly after the addition of FA. At an FA dose of 0.9 g, the Trp band intensity increased by 36.07%, indicating that Trp residues were located in a more hydrophobic and spatially restricted microenvironment, which may be associated with local molecular association and conformational stabilization. Overall, the results showed that FA-induced changes in the microenvironments of Tyr and Trp residues were strongly dependent on protein type and matrix conditions. In the dough system, Trp residues in glutenin tended to be located in a more hydrophobic and restricted microenvironment, whereas in glutenin isolated from the simulated system, Trp residues were more exposed to a polar environment. Gliadin showed complex dose-dependent changes in the dough system, but tended to undergo local hydrophobic association in the simulated system. These patterns further indicated that FA regulated the tertiary conformations of glutenin and gliadin through different pathways in the two systems.

#### 3.5.2. Disulfide Bond Conformation

Disulfide bonds are the primary mode of intermolecular covalent aggregation in proteins, exerting a significant influence on protein structure [49]. Based on the varying C-S-S-C atomic configurations, disulfide bond bridges can be classified into gauche–gauche–gauche (g-g-g), trans–gauche–gauche (t-g-g), and trans–gauche–trans (t-g-t) conformations [50]. Among these, the g-g-g conformation is the most stable. The disulfide bond conformations of the protein are shown in Table 1. The deconvoluted bands corresponding to the disulfide bond conformations are shown in Figures S7–S10. In the dough system, the proportion of the g-g-g conformation of glutenin changed little overall, but it decreased at low to medium FA doses, while the t-g-g and t-g-t conformations increased under different treatments. At an FA dose of 0.9 g, the proportion of the g-g-g conformation decreased by 10.67% compared to the control group. This indicated that FA reduced the stability of disulfide bonds in glutenin. This was in agreement with the study by Han & Koh [51], which reported that the reducing property of FA could disrupt the intermolecular disulfide bonds of proteins, thereby weakening the gluten network structure. However, at higher FA doses, glutenin underwent a certain degree of re-aggregation within the dough environment. For gliadin in the dough system, the proportion of the g-g-g conformation continuously increased with the addition of FA, whereas the proportion of the t-g-t conformation decreased significantly. This suggested that FA generally facilitated the transition of disulfide bond conformations in gliadin toward a stable state. Previous studies have shown that high doses of FA can cause excessive aggregation of gluten proteins in dough [52]. The increase in the proportion of g-g-g conformation of glutenin and gliadin at high doses suggested that FA might have induced further covalent binding of gluten proteins. In the simulated dough system, however, the proportion of the g-g-g conformation in glutenin decreased significantly after the addition of FA, while that of the t-g-t conformation increased markedly. This suggested that when FA acted directly on isolated glutenin, it disturbed the original stable disulfide bond conformations and reduced their structural stability. In the simulated system lacking starch and the original gluten network constraint, FA was more likely to directly act on the glutenin molecules, disrupting the conformational balance around the original disulfide bonds, which led to the unfolding of protein chains and the loosening of local structures. Although the proportion of the g-g-g conformation slightly increased at high doses, it was still significantly lower than that of the control, indicating that the disulfide bond rearrangement induced by FA in the glutenin of the simulated system was stronger and less reversible. In contrast, the proportion of the g-g-g conformation in gliadin increased significantly with the addition of FA in the simulated dough system. When 1.5 g of FA was added, the proportion of the g-g-g conformation rose to 55.14%, an increase of 42.89% compared to the control group. Glutenin is a linear polymer connected by intermolecular disulfide bonds, so direct FA treatment might have more readily disrupted its original network and shifted the stable g-g-g conformation toward less stable forms. Gliadin, by comparison, is a monomeric protein rich in intramolecular disulfide bonds and has a relatively loose structure, which makes it more susceptible to conformational changes [49]. This was consistent with the research of Lin et al. [18], where under FA treatment, gliadin underwent two-stage changes: initially aggregating into larger clusters, and then solidifying into denser aggregates.

### 3.6. Effects of FA on Chemical Interactions of Glutenin and Gliadin

**Figure 5:**
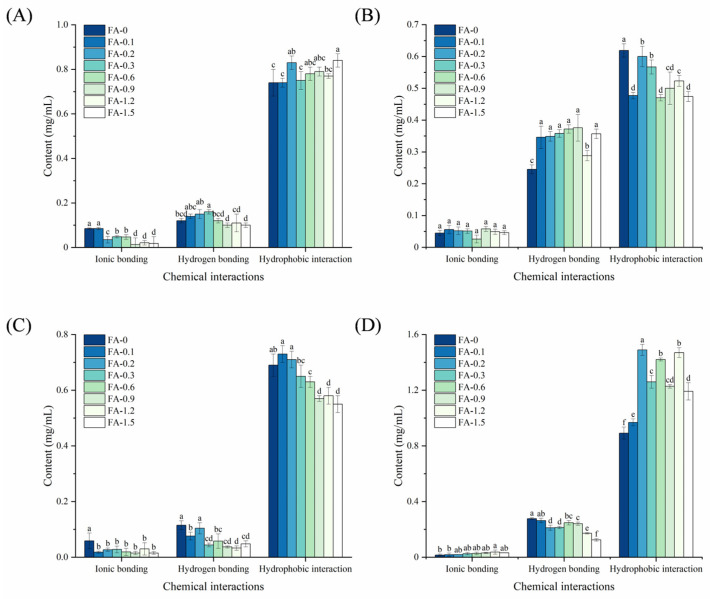
Effects of FA on chemical interactions of glutenin and gliadin: (**A**) glutenin in the dough system; (**B**) gliadin in the dough system; (**C**) glutenin in the simulated dough system; (**D**) gliadin in the simulated dough system. Different lowercase letters within a column denote significant difference at *p* < 0.05.

Intermolecular interactions among protein molecules are important for gluten network formation and together determine the development of the gluten structures [53]. The contributions of the three non-covalent chemical interactions (ionic bonds, hydrogen bonds, and hydrophobic interactions) that maintain glutenin and gliadin are shown in Figure 5. The results indicated that hydrophobic interactions constituted the predominant non-covalent forces maintaining the gluten protein components. Previous research has pointed out that the initial phase of phenolic acid–gluten interactions is mediated by ionic and hydrogen bonds, whereas hydrophobic interactions govern their specificity [18]. In the dough system, the hydrophobic interactions of glutenin increased with the addition of FA and were consistently maintained at a high level. This could be attributed to the fact that the addition of FA disrupted the gluten protein structure, leading to the exposure of hydrophobic groups. Furthermore, the enhancement of hydrophobic interactions would drive the hydrophobic regions on the surfaces of initially dispersed protein molecules to bind more intensely with one another, thereby promoting the aggregation of glutenin. This agrees with Lin et al. [18], who suggested that FA could promote glutenin cross-linking through interactions with hydrophobic regions. In contrast, gliadin in the dough system showed the opposite trend. This might be because gliadin is rich in glutamine and proline residues, while FA contains phenolic hydroxyl groups, which favor hydrogen bond formation between them. FA bound to the surface of gliadin via hydrogen bonds; given the inherent polarity of FA itself, the hydrophobic interactions were consequently masked [54]. Therefore, it was observed that following the addition of FA, the hydrogen bonds of gliadin in the dough system were strengthened, whereas the hydrophobic interactions were disrupted. This indicated that FA had a higher tendency to reinforce the hydrogen-bonding network and alter the local hydrophobic equilibrium, which was consistent with the results reported by Lin et al. [18] regarding the FA-induced enhancement of the hydrogen-bonding network in gliadin. The non-covalent interactions of glutenin and gliadin in the simulated dough system exhibited significant differences compared to those in the dough system. Consistent with the dough system, the ionic bond content of glutenin in the simulated system showed a decreasing trend. This might be attributed to the acidic nature of FA, which altered the charge distribution in the microenvironment of glutenin, resulting in the disruption of the electrostatic interactions that maintain its tertiary structure [55]. Regarding hydrogen bonds, the content in dough-system glutenin slightly increased at low FA doses; however, in the simulated dough system, due to the absence of buffering effects from starch and other components, FA directly occupied the hydrogen-bonding sites, thereby disrupting the internal hydrogen bonds of glutenin. Furthermore, the impact of FA on the hydrophobic interactions of glutenin and gliadin in simulated dough showed significant differences. After the addition of FA, the hydrophobic interactions of glutenin in the simulated dough system decreased. When FA acted directly on glutenin, it likely disturbed the hydrophobic packing of the protein, thereby weakening the hydrophobic interactions that helped stabilize the aggregates. Conversely, gliadin is a monomeric protein with a low molecular weight but is exceedingly hydrophobic. In the simulated dough system, FA forcibly inserted into the interior of gliadin via hydrogen bonds, which instantly exposed the large hydrophobic core inside gliadin [56]. The exposed hydrophobic groups subsequently underwent hydrophobic reaggregation to form more compact aggregates. Accordingly, the hydrophobic interaction of gliadin in the simulated dough system supplemented with 1.2 g FA increased by 64.8% compared with the control group, which was consistent with the results of fluorescence spectroscopy in this study.

### 3.7. Effects of FA on the Free Sulfhydryl Group Content of Glutenin and Gliadin

**Figure 6:**
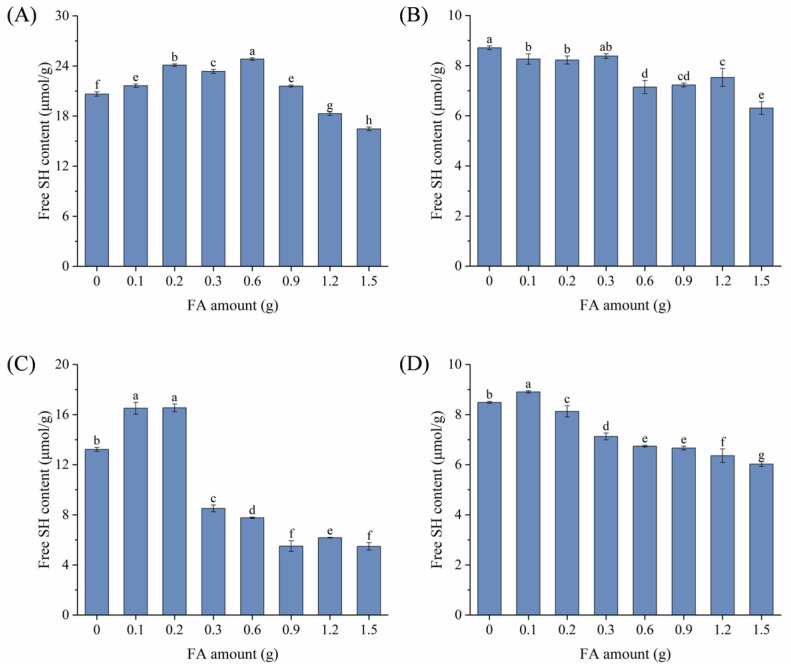
Effects of FA on the free sulfhydryl group content of glutenin and gliadin: (**A**) glutenin in the dough system; (**B**) gliadin in the dough system; (**C**) glutenin in the simulated dough system; (**D**) gliadin in the simulated dough system. Different lowercase letters within a column denote significant difference at *p* < 0.05.

The exchange reaction between free sulfhydryl groups and disulfide bonds plays a crucial role in maintaining the structure and function of gluten proteins; consequently, the free sulfhydryl group content can be utilized to evaluate the aggregation state and molecular structure of gluten [57]. In the dough system, when 0.6 g of FA was added, the free sulfhydryl group content of glutenin increased significantly compared to the control group. However, when the addition of FA exceeded 0.6 g, the free sulfhydryl group content began to decline (Figure 6A). The reducing property of FA potentially disrupted the disulfide bonds of glutenin, leading to the release of free sulfhydryl groups without complete recombination. The reducing capacity of FA may disrupt disulfide bonds in glutenin, leading to the release of free sulfhydryl groups before complete disulfide bond reformation. This increase in free sulfhydryl group content suggested that the glutenin aggregates became partially loosened [58]. However, with the further addition of FA, the quinone intermediates generated from FA oxidation acted as “molecular bridges,” forming covalent bonds with the free sulfhydryl groups on the proteins, thereby facilitating the development of a cross-linking network [51]. Furthermore, the depolymerized proteins might have provided additional binding sites, inducing protein re-aggregation and consequently leading to a decrease in the free sulfhydryl group content. In contrast, the free sulfhydryl group content of gliadin progressively decreased with the addition of FA, and the magnitude of change was less pronounced than that observed in glutenin (Figure 6B). Because gliadin contains less cysteine and mainly forms intramolecular disulfide bonds, its disulfide linkages are less easily affected by FA. In addition, the relatively loose structure of gliadin might have allowed FA to react more readily with exposed free sulfhydryl groups, thereby promoting disulfide bond rearrangement. A similar phenomenon was reported by Wang et al. [13], who found that FA promoted gluten aggregation in steamed bread. By contrast, the changes in glutenin in the simulated dough system were more evident (Figure 6C). Low-dose FA results in an increase in the content of free sulfhydryl groups. This suggests that when FA directly interacts with isolated glutenin, it can disrupt the conformation of glutenin and expose free sulfhydryl groups. When the FA dosage exceeded 0.2 g, the content of free sulfhydryl groups decreased rapidly. At high concentrations, FA came into direct contact with glutenin, and the active double bonds of FA might have formed C-S bonds with cystine, affecting the mutual conversion between sulfhydryl groups and disulfide bonds [39]. Therefore, even though the sulfhydryl group content decreased, the glutenin in the simulated dough system did not aggregate, which was consistent with the decrease in the proportion of the g-g-g conformation of disulfide bonds in this study. The variation trend of free sulfhydryl group content of gliadin in the simulated dough system (Figure 6D) was consistent with that of glutenin, but gliadin was less sensitive to the effect of FA than glutenin.

### 3.8. Effects of FA on the Subunit Content of Glutenin and Gliadin

To clarify the effects of FA on the cross-linking and polymerization of glutenin and gliadin, the extraction rates of protein subunits in different systems were analyzed by RP-HPLC. Table 2 shows that the response of gluten proteins to FA differed between systems. In the dough system, the extraction rates of the three glutenin subunits all showed an overall decrease. This might have been because FA promoted the rearrangement of glutenin in the dough, resulting in the formation of larger aggregates and a lower subunit extraction rate. Among these subunits, B/C-LMW-GS showed the largest decrease after the addition of FA. In particular, when 1.5 g of FA was added, the extraction rate of B/C-LMW-GS decreased by 25.43% compared to the control group. The magnitude of change for D-LMW-GS was relatively small, and HMW-GS also exhibited an overall downward trend, although it rebounded slightly when 0.9 g of FA was added. This indicated that B/C-LMW-GS may have preferentially participated in the formation of larger aggregates within the dough, and by further affecting HMW-GS, it propelled glutenin from an initial rearrangement toward stronger aggregation [18]. For gliadin in the dough system, the peak areas of ω-gliadin and α-gliadin generally increased. In contrast, γ-gliadin decreased at medium FA doses, but rebounded when 1.2 g of FA was added. Previous studies have indicated that gliadin exhibits excellent solubility and dispersibility at low pH values [59]. However, a higher content of gliadin nanoparticles in the solution increases the probability of forming aggregated structures [60]. Therefore, the increased solubility of gliadin caused by the acidic nature of FA might also have facilitated the formation of gluten aggregates. Wang et al. [16] reported that FA reduced the extraction rate of B/C-LMW-GS and increased the extraction rate of gliadin subunits in steamed bread. FA promoted gluten aggregation in steamed bread, thereby leading to a deterioration in its quality. This was consistent with the results of the present study. By contrast, the peak areas of the three glutenin subunits—D-LMW-GS, HMW-GS, and B/C-LMW-GS—in the simulated dough system all exhibited an overall upward trend with the addition of FA. This suggested that in the absence of media such as starch, the direct effect of FA on isolated glutenin initially led to the unfolding of chain segments and the exposure of reactive groups, thereby enhancing the extractability of the subunits. For gliadin in the simulated dough system, the peak areas of ω-gliadin and γ-gliadin generally decreased, whereas α-gliadin exhibited an initial increase followed by a decrease. This indicated discrepancies in the direct effects of FA on gliadin. α-Gliadin was more prone to conformational loosening and exposure at low to medium doses, and subsequently entered a state of aggregation or burial at high doses. Conversely, ω-gliadin and γ-gliadin tended to continuously participate in the aggregation process. These results suggested that FA not only affected the content of individual subunits, but also influenced the conformational changes of gluten proteins by altering the involvement of different subunits in aggregation, retention, and extraction.

### 3.9. Effects of Ferulic Acid on the Molecular Weight Distribution of Glutenin and Gliadin

**Figure 7:**
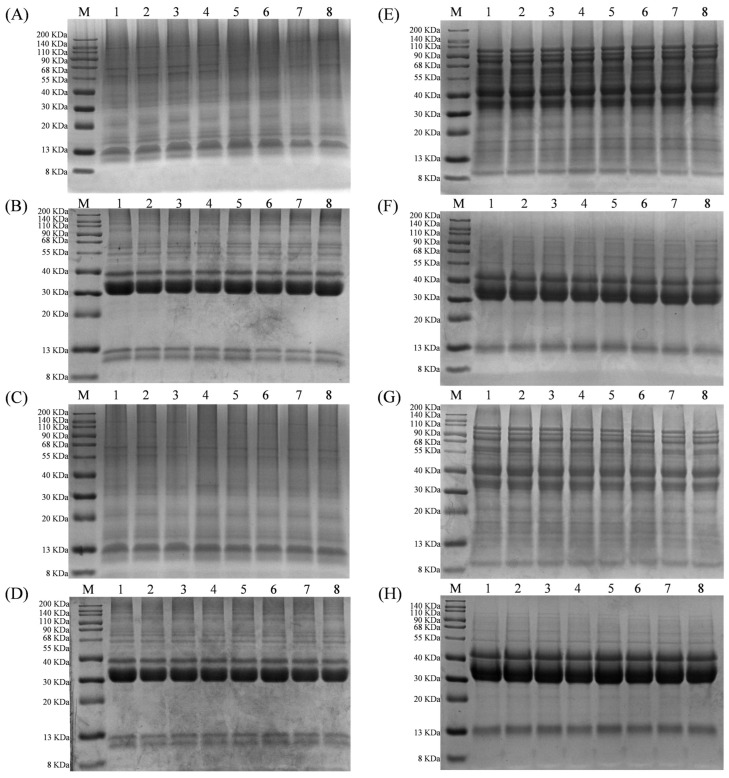
Effects of FA on the electrophoretic band patterns of glutenin and gliadin: (**A**–**D**) show the electrophoretic bands under non-reducing conditions, and (**E**–**H**) show the electrophoretic bands under reducing conditions. (**A**,**E**) Glutenin in the dough system; (**B**,**F**) gliadin in the dough system; (**C**,**G**) glutenin in the simulated dough system; (**D**,**H**) gliadin in the simulated dough system. M, protein marker; lanes 1–8, proteins treated with 0, 0.1, 0.2, 0.3, 0.6, 0.9, 1.2, and 1.5 g FA, respectively.

The electrophoretic patterns of glutenin and gliadin under non-reducing and reducing conditions were analyzed to further evaluate whether FA altered the aggregation state and disulfide bond-related structures of these proteins. As shown in Figure 7, no obvious changes were observed in the number or position of electrophoretic bands of glutenin and gliadin among the different FA treatments, indicating that the addition of FA did not change the subunit composition of glutenin or gliadin. For glutenin in the dough system under non-reducing conditions, the low-molecular-weight bands were markedly intensified at low FA doses (≤0.3 g), suggesting partial loosening of glutenin aggregates. At higher FA doses, the original characteristic bands became weaker and more diffuse, indicating that some glutenin subunits may have formed larger and more compact aggregates with lower electrophoretic mobility. In contrast, clearer glutenin subunit bands were observed under reducing conditions, confirming that the macromolecular aggregates with delayed migration in non-reducing SDS-PAGE were partly generated through disulfide bond-mediated cross-linking. Compared with glutenin, gliadin in the dough system showed relatively slight differences between reducing and non-reducing electrophoretic patterns. No obvious accumulation of high-molecular-weight polymers was observed in the gliadin bands. This suggested that FA-induced structural changes in gliadin did not mainly depend on intermolecular disulfide bond cross-linking, but were more closely aggregated with non-covalent interactions. This result was consistent with previous findings that gliadin mainly forms intramolecular disulfide bonds [48]. In the simulated protein system, FA also changed the band intensity and subunit migration behavior of the proteins, with a more pronounced effect on glutenin. However, high-molecular-weight polymer bands similar to those observed for glutenin in the dough system were not formed. These results indicated that when FA directly interacted with isolated glutenin, it mainly promoted protein structural loosening and disulfide bond conformational rearrangement, but was insufficient to form an extensive disulfide-linked covalent cross-linking network.

### 3.10. Molecular Docking Study of FA with Glutenin and Gliadin

**Figure 8:**
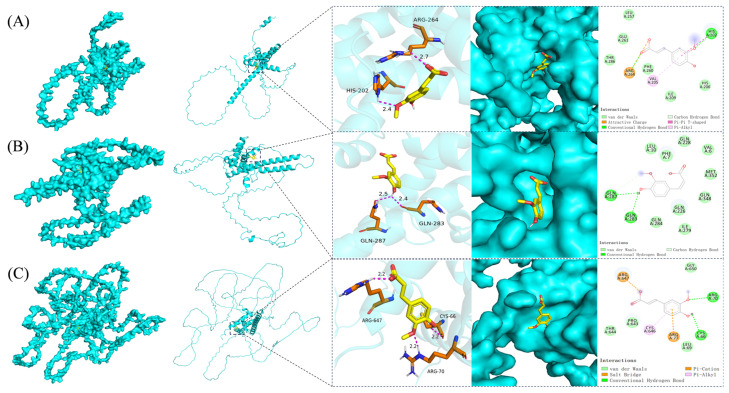
Molecular docking study of FA with glutenin and gliadin. (**A**) Gliadin; (**B**) low-molecular-weight glutenin; (**C**) high-molecular-weight glutenin (In the two-dimensional interaction diagram shown in panel (**C**), the two orange lines represent different interactions: the orange line connecting oppositely charged groups indicates a salt bridge, whereas the orange line terminating at the center of the aromatic ring indicates a π–cation interaction).

To further explore the possible molecular basis for the interaction of FA with glutenin and gliadin, molecular docking was carried out based on the model reported by Du et al. [35]. The results are shown in Figure 8. The docking results revealed the potential binding modes and binding sites of FA with gliadin, LMW-GS, and HMW-GS. The predicted binding energies of FA with gliadin, LMW-GS, and HMW-GS were −6.0, −5.7, and −5.3 kcal/mol, respectively. Among them, FA showed the strongest predicted affinity for gliadin. In the gliadin model, FA was predicted to be accommodated in a local groove-like region on the protein surface, where it formed hydrogen bonds with His-202 and Arg-264. In addition, electrostatic interactions, π–π/π–alkyl interactions, and van der Waals forces with surrounding residues may have contributed to the stabilization of this binding pose. Since gliadin is a globular monomeric protein mainly stabilized by intramolecular disulfide bonds, its local hydrophobic regions and aromatic-residue microenvironment may be relatively sensitive to small-molecule interactions [18]. Thus, the predicted binding mode of FA with gliadin is consistent with the fluorescence and Raman results, including fluorescence quenching and red shift, decreased Trp band intensity, and increased Tyr doublet ratio. These results suggest that FA may preferentially affect the local microenvironment of gliadin, especially around aromatic residues and hydrogen-bonding regions. The interaction between FA and LMW-GS mainly relied on a dense hydrogen-bond network formed between its hydroxyl/carboxyl groups and glutamine-rich residues (Gln-283, Gln-287). This was consistent with the trend of variation in hydrogen bond content in the chemical interaction analysis. This indicated that FA was more likely to preferentially affect the hydrogen bond network and local chain segment arrangement of LMW-GS, which was also consistent with the fact that the LMW component changes were more sensitive in the subunit analysis. In contrast, FA showed a relatively weaker predicted binding affinity toward HMW-GS. However, the predicted binding region was located near Arg- and Cys-adjacent sites. The side chain of Arg is positively charged; it can not only form electrostatic attraction with the carboxyl group of FA but also provide hydrogen bonds, thus strengthening the directional binding of the ligand at this site [61]. Cys, on the other hand, is closely related to the sulfhydryl groups/disulfide bond status [62]. The binding of FA to its neighboring region might have further affected sulfhydryl group exposure or disulfide exchange by altering the local spatial conformation and microenvironment. This indicated that although FA did not bind to HMW-GS with the strongest affinity, it most likely regulated the local charge environment, hydrophobic packing, and sulfhydryl group/disulfide bond-related structures of HMW-GS via key sites, further leading to the rearrangement of the entire glutenin network. Molecular docking was used as a supplementary method to infer the possible binding modes between FA and different gluten protein components. The results showed that FA could spontaneously bind to glutenin and gliadin, and targeted key sites of different proteins through hydrogen bonds, electrostatic interactions, hydrophobic interactions and other forces, thereby perturbing the protein hydrophobic core, hydrogen bond network, and sulfhydryl group/disulfide bond equilibrium to exert effects on protein structure. It should be noted that molecular docking calculations, based on static protein crystal structures or homology models, cannot fully simulate the heterogeneous distribution of starch, lipids, and water in real dough, nor the combined effects of dynamic processing conditions on the interactions between FA and gluten protein components. Therefore, molecular docking results serve as a supplementary interpretive tool at the structural level, providing a visual explanation of the spatial conformations underlying experimental phenomena. From the perspective of intermolecular forces, this analysis provides a reasonable hypothesis for the differences in binding affinity and binding sites between gliadin and glutenin, and offers a directional reference for subsequent, more in-depth research. In this study, molecular docking analysis complements experimental results such as fluorescence spectroscopy, Raman spectroscopy, and free thiol assays, collectively supporting the interaction mechanism between FA and gluten protein components.

## 4. Discussion

This study systematically investigated the effects of FA on glutenin and gliadin under different interaction systems, with emphasis on aggregation behavior, spatial conformation, intermolecular interactions, and molecular docking. The results showed that the effects of FA on glutenin and gliadin were strongly dose-dependent and system-dependent. In glutenin from the dough system, a low FA dose (0.3 g) perturbed the surface charge environment, promoted the transformation of β-sheets into β-turns and random coils, and disrupted disulfide bonds through the reducing capacity of FA. Meanwhile, the increased proportion of unstable disulfide bond conformations and subunit rearrangement contributed to protein loosening, as reflected by the decrease in particle size. However, with increasing FA dose, FA promoted the re-aggregation of glutenin through local microenvironment remodeling, hydrophobic aggregation, strengthening of hydrogen-bonding networks, and covalent cross-linking, thereby forming more compact aggregates and increasing particle size. In gliadin from the dough system, low-dose FA was first adsorbed onto the protein surface and polar sites through ionic interactions and hydrogen bonding, disrupting the local charge environment and hydrogen-bonding balance. FA also disturbed the tertiary structure of gliadin, leading to the exposure of hydrophobic groups. At high FA doses, the exposed hydrophobic regions began to re-participate in intermolecular aggregations, while the newly formed hydrogen-bonding network and enhanced covalent cross-linking further promoted protein aggregation. In contrast, FA acted more directly on isolated glutenin and gliadin in the simulated system. FA directly disturbed the hydrophobic regions of glutenin, disrupted its tertiary structure, and weakened the hydrogen-bonding network and hydrophobic interactions, resulting in protein unfolding. In gliadin from the simulated system, the increased proportion of the g-g-g disulfide bond conformation and the decreased subunit extraction rate promoted protein aggregation and further contributed to the formation of larger or insoluble aggregates. The effects of FA on glutenin and gliadin in the dough system observed in this study were consistent with previous reports showing that FA promoted gluten protein aggregation in steamed bread [13]. Based on these findings, we further explored the underlying mechanisms involved. Moreover, this study provided deeper insight into the effects of FA on isolated glutenin and gliadin.

The obvious differences between the dough system and the simulated dough system were mainly attributed to the complex multiphase coupling environment formed by starch, proteins, and water in real dough. According to the reports of previous studies, starch not only competed with proteins for water and restricted the free hydration and full unfolding of protein chains, but also changed the approach and aggregation pathways of protein chains through physical filling [63]. Therefore, in real dough, FA was more likely to induce rearrangement and re-aggregation of the pre-existing gluten network, rather than directly causing local exposure and conformational destabilization of isolated proteins as observed in the simulated system. In addition, starch further regulated the intensity of FA–protein interactions by affecting water migration and the state of bound water [64]. Thus, the effect of FA in the dough system essentially reflected the overall regulation of the starch–protein–water ternary system, whereas the simulated dough system mainly reflected the direct structural effects of FA on the proteins themselves. However, the specific regulatory mechanisms of FA on gluten proteins and their components in complex dough systems still require further investigation. Moreover, because starch is the most abundant component in wheat flour, the effects of FA on starch also need to be further explored.

This study further clarified the FA-induced molecular structural changes in glutenin and gliadin, which may subsequently affect dough processing properties and the quality of wheat-based products. Glutenin mainly contributes to dough strength and elasticity, whereas gliadin is closely related to viscosity and extensibility [22,23]. In the dough system, low-dose FA caused a decrease in particle size, partial relaxation of secondary structure, an increase in free sulfhydryl group content, and changes in disulfide bond conformations, indicating that glutenin became more dispersed and flexible. This may improve dough extensibility to some extent. In contrast, high-dose FA promoted glutenin aggregation and formed a more compact glutenin structure. Such excessive aggregation restricted the mobility of protein chains and may reduce dough extensibility. For gliadin, FA-induced changes in the local microenvironment, hydrogen bonding, hydrophobic interactions, and subunit extraction rate may affect dough viscosity, rheological behavior, and the ability of wheat gluten proteins to form a continuous network. In particular, excessive aggregation of gliadin may weaken its plasticizing effect on the gluten network, thereby affecting dough extensibility and processing tolerance. Therefore, low-dose FA may improve certain aspects of dough and wheat-based product quality, whereas excessive aggregation of glutenin and gliadin induced by high-dose FA may further hinder dough proofing and expansion, ultimately reducing the quality of wheat-based products. This further confirmed previous findings that FA affected the quality of wheat-based products, such as steamed bread and bread, in a dose-dependent manner [13].

## 5. Conclusions

The results showed that the interactions between FA and gluten protein components strongly depended on FA dose, protein type, and system environment. In the dough system, FA induced a transition in glutenin and gliadin from structural loosening at low doses to re-aggregation at high doses. In the simulated system, FA acted more directly on the isolated protein components, leading to stronger structural loosening of glutenin, whereas gliadin showed more pronounced aggregation and stabilization. These differences suggested that starch, lipids, water distribution, and the pre-existing gluten network in the real dough matrix may regulate or alter the direct FA–protein interactions observed in the simplified protein system. These findings deepen our understanding of how FA regulates glutenin and gliadin in a dose- and system-dependent manner. Meanwhile, FA may affect dough processing quality by altering the balance among protein loosening, aggregation, and network rearrangement. This study elucidated the interaction mechanisms between FA and wheat protein components in different systems, providing theoretical support for understanding the role of FA in wheat-based food systems. However, the effects of FA on starch still require further investigation to fully clarify the overall influence of FA on wheat dough quality.

## Figures and Tables

**Table 1:** Effects of FA on the amino acid side chains and disulfide bond conformation of glutenin and gliadin.

Protein Type	FA Amount (g)	I[850]/I[830]	I[760]	SS ggg (%)	SS tgg (%)	SS tgt (%)
Glutenin in the dough system	0	1.44 ± 0.04 ^a^	1.27 ± 0.03 ^f^	44.50 ± 0.80 ^a^	28.86 ± 1.58 ^c^	26.63 ± 0.96 ^f^
	0.1	1.03 ± 0.08 ^c^	1.72 ± 0.01 ^e^	43.67 ± 0.57 ^abc^	24.28 ± 0.14 ^e^	32.04 ± 0.63 ^ab^
	0.2	0.95 ± 0.02 ^cd^	2.15 ± 0.02 ^bc^	42.94 ± 0.52 ^cd^	25.81 ± 0.58 ^d^	31.25 ± 0.14 ^bc^
	0.3	1.25 ± 0.05 ^b^	1.81 ± 0.00 ^de^	43.91 ± 0.11 ^ab^	25.15 ± 0.04 ^de^	30.95 ± 0.06 ^c^
	0.6	1.00 ± 0.07 ^cd^	2.15 ± 0.01 ^bc^	43.14 ± 3.01 ^bcd^	33.68 ± 0.01 ^a^	23.18 ± 0.01 ^g^
	0.9	0.92 ± 0.02 ^d^	1.98 ± 0.29 ^dc^	39.75 ± 0.05 ^e^	31.13 ± 0.09 ^b^	29.11 ± 0.03 ^d^
	1.2	1.02 ± 0.04 ^c^	2.52 ± 0.15 ^a^	44.02 ± 0.69 ^ab^	28.42 ± 0.09 ^c^	27.56 ± 0.78 ^e^
	1.5	1.00 ± 0.04 ^cd^	2.30 ± 0.07 ^b^	42.72 ± 0.19 ^d^	24.71 ± 0.43 ^de^	32.57 ± 0.25 ^a^
Gliadin in the dough system	0	0.87 ± 0.02 ^d^	1.54 ± 0.02 ^d^	31.65 ± 0.54 ^g^	14.72 ± 0.16 ^e^	53.63 ± 0.37 ^ab^
	0.1	0.97 ± 0.03 ^c^	1.66 ± 0.03 ^bc^	32.57 ± 0.58 ^fg^	18.47 ± 1.27 ^d^	48.96 ± 0.99 ^b^
	0.2	0.85 ± 0.01 ^d^	1.86 ± 0.03 ^a^	35.00 ± 0.41 ^e^	23.50 ± 1.05 ^c^	41.50 ± 1.12 ^c^
	0.3	0.76 ± 0.08 ^e^	1.24 ± 0.00 ^f^	33.49 ± 0.30 ^f^	10.25 ± 0.42 ^f^	56.26 ± 0.12 ^a^
	0.6	0.98 ± 0.06 ^c^	1.74 ± 0.17 ^b^	37.25 ± 0.11 ^d^	42.22 ± 0.99 ^a^	23.87 ± 6.70 ^d^
	0.9	1.07 ± 0.00 ^ab^	1.63 ± 0.01 ^cd^	48.22 ± 0.13 ^b^	34.97 ± 3.73 ^b^	16.82 ± 3.61 ^e^
	1.2	0.99 ± 0.03 ^bc^	1.41 ± 0.02 ^e^	42.38 ± 1.15 ^c^	35.57 ± 0.83 ^b^	22.06 ± 1.24 ^d^
	1.5	1.10 ± 0.08 ^a^	1.38 ± 0.01 ^e^	50.91 ± 0.45 ^a^	37.61 ± 0.45 ^b^	11.48 ± 0.01 ^f^
Glutenin in the simulated dough system	0	1.13 ± 0.00 ^a^	2.67 ± 0.02 ^a^	50.87 ± 0.06 ^a^	35.49 ± 0.39 ^a^	13.64 ± 0.32 ^e^
	0.1	1.15 ± 0.03 ^a^	1.31 ± 0.1 ^g^	46.94 ± 0.71 ^b^	31.63 ± 0.70 ^cd^	21.43 ± 0.09 ^d^
	0.2	1.03 ± 0.03 ^b^	1.33 ± 0.01 ^g^	44.19 ± 0.95 ^c^	34.67 ± 1.23 ^ab^	21.14 ± 1.49 ^d^
	0.3	1.00 ± 0.01 ^b^	2.09 ± 0.04 ^c^	42.92 ± 1.62 ^c^	32.85 ± 1.35 ^bc^	24.23 ± 2.97 ^cd^
	0.6	0.85 ± 0.04 ^c^	1.73 ± 0.00 ^e^	42.72 ± 0.28 ^c^	29.87 ± 1.93 ^de^	27.41 ± 1.90 ^c^
	0.9	1.01 ± 0.02 ^b^	1.46 ± 0.01 ^f^	37.40 ± 3.25 ^de^	13.22 ± 0.17 ^f^	49.38 ± 3.42 ^a^
	1.2	1.03 ± 0.1 ^b^	1.89 ± 0.06 ^d^	35.76 ± 0.14 ^e^	32.89 ± 0.79 ^bc^	31.35 ± 0.65 ^b^
	1.5	0.97 ± 0.06 ^b^	2.50 ± 0.05 ^b^	38.76 ± 0.18 ^d^	29.29 ± 0.27 ^e^	31.94 ± 0.45 ^b^
Gliadin in the simulated dough system	0	0.99 ± 0.09 ^c^	1.83 ± 0.03 ^d^	38.59 ± 0.38 ^e^	21.69 ± 0.85 ^e^	39.72 ± 0.47 ^a^
	0.1	1.00 ± 0.08 ^c^	2.14 ± 0.02 ^c^	39.11 ± 0.80 ^cde^	25.75 ± 1.96 ^e^	35.13 ± 2.75 ^b^
	0.2	0.82 ± 0.02 ^d^	4.13 ± 0.28 ^a^	40.24 ± 0.01 ^c^	32.40 ± 0.09 ^d^	27.36 ± 0.09 ^c^
	0.3	1.26 ± 0.04 ^a^	2.32 ± 0.21 ^bc^	38.79 ± 0.26 ^de^	33.99 ± 2.99 ^cd^	27.22 ± 2.72 ^c^
	0.6	0.96 ± 0.09 ^c^	2.13 ± 0.02 ^c^	43.61 ± 0.57 ^b^	40.50 ± 4.97 ^ab^	15.89 ± 4.40 ^d^
	0.9	1.12 ± 0.07 ^b^	2.49 ± 0.31 ^b^	39.88 ± 0.97 ^cd^	44.77 ± 1.67 ^a^	15.35 ± 0.70 ^d^
	1.2	0.91 ± 0.05 ^cd^	2.14 ± 0.00 ^c^	43.56 ± 0.81 ^b^	40.37 ± 0.07 ^a^	16.08 ± 0.88 ^d^
	1.5	0.95 ± 0.04 ^c^	2.22 ± 0.00 ^bc^	55.14 ± 0.97 ^a^	37.51 ± 2.88 ^bc^	7.35 ± 2.23 ^e^

Results represent mean ± standard deviation (*n* = 3). Means in the same column with different lowercase letter superscripts indicate significant difference (*p* < 0.05).

**Table 2:** Effects of FA on the subunit content of glutenin and gliadin.

Protein System	FA Amount (g)	Glutenin			Gliadin		
		D-LMW-GS (mAU, 1 × 10^5^)	HMW-GS (mAU, 1 × 10^5^)	B/C-LMW-GS (mAU, 1 × 10^5^)	ω-Gliadin (mAU, 1 × 10^5^)	α-Gliadin (mAU, 1 × 10^5^)	γ-Gliadin (mAU, 1 × 10^5^)
Dough system	0	144.62 ± 3.18 ^a^	573.23 ± 3.16 ^b^	1191.25 ± 36.95 ^a^	851.78 ± 35.97 ^d^	854.76 ± 30.48 ^e^	839.89 ± 21.62 ^b^
	0.1	134.25 ± 3.45 ^ab^	562.33 ± 5.32 ^bc^	1102.11 ± 10.30 ^b^	862.47 ± 31.77 ^d^	896.07 ± 10.87 ^de^	809.94 ± 19.64 ^bc^
	0.2	132.35 ± 4.32 ^ab^	532.43 ± 4.23 ^d^	1065.34 ± 7.95 ^bc^	857.84 ± 13.97 ^d^	921.98 ± 19.21 ^cd^	814.96 ± 23.63 ^bc^
	0.3	129.67 ± 9.20 ^b^	511.64 ± 3.16 ^e^	1010.13 ± 10.48 ^d^	882.98 ± 1.14 ^cd^	966.48 ± 19.40 ^c^	777.01 ± 3.90 ^c^
	0.6	134.66 ± 1.04 ^ab^	542.80 ± 23.80 ^cd^	1046.86 ± 40.38 ^cd^	891.86 ± 35.10 ^cd^	953.75 ± 39.30 ^c^	832.55 ± 20.01 ^b^
	0.9	133.80 ± 14.12 ^ab^	605.96 ± 5.86 ^a^	1023.94 ± 4.75 ^d^	939.02 ± 18.59 ^bc^	1025.96 ± 9.87 ^b^	766.64 ± 41.03 ^c^
	1.2	138.06 ± 10.40 ^ab^	501.88 ± 5.02 ^e^	949.51 ± 18.91 ^e^	1012.52 ± 25.20 ^a^	1079.34 ± 19.10 ^a^	902.64 ± 32.32 ^a^
	1.5	143.79 ± 4.35 ^ab^	444.23 ± 10.23 ^f^	888.31 ± 7.41 ^f^	976.68 ± 57.64 ^ab^	1043.72 ± 38.97 ^ab^	778.77 ± 44.31 ^c^
Simulated dough system	0	173.70 ± 11.21 ^f^	578.07 ± 12.46 ^de^	1021.75 ± 20.37 ^e^	886.98 ± 24.24 ^a^	1006.60 ± 17.29 ^e^	1125.26 ± 2.07 ^a^
	0.1	180.83 ± 9.56 ^ef^	591.23 ± 7.88 ^cd^	1033.64 ± 10.46 ^e^	790.35 ± 10.83 ^b^	1020.34 ± 10.34 ^e^	1070.74 ± 10.35 ^b^
	0.2	192.98 ± 7.63 ^de^	607.33 ± 5.64 ^c^	1098.98 ± 11.45 ^d^	774.88 ± 17.32 ^b^	1102.45 ± 15.32 ^c^	1045.43 ± 6.32 ^b^
	0.3	210.49 ± 9.92 ^c^	600.42 ± 11.36 ^c^	1162.64 ± 42.18 ^c^	690.79 ± 45.50 ^c^	1184.61 ± 18.89 ^a^	1028.84 ± 21.67 ^b^
	0.6	199.48 ± 4.42 ^cd^	561.23 ± 9.66 ^e^	1184.21 ± 8.97 ^c^	709.35 ± 10.97 ^c^	1194.65 ± 13.46 ^a^	968.81 ± 6.59 ^c^
	0.9	228.04 ± 14.16 ^b^	640.18 ± 9.86 ^b^	1305.42 ± 17.85 ^b^	681.49 ± 16.50 ^c^	1143.69 ± 36.13 ^b^	969.24 ± 34.40 ^c^
	1.2	252.86 ± 8.74 ^a^	649.07 ± 3.65 ^b^	1267.64 ± 13.35 ^b^	629.99 ± 10.42 ^d^	1063.09 ± 24.07 ^d^	965.34 ± 49.43 ^c^
	1.5	249.77 ± 3.19 ^a^	681.44 ± 16.31 ^a^	1420.85 ± 71.34 ^a^	633.64 ± 12.00 ^d^	835.46 ± 16.57 ^f^	920.57 ± 17.04 ^d^

Results represent mean ± standard deviation (*n* = 3). Means in the same column with different lowercase letter superscripts indicate significant difference (*p* < 0.05).

## Data Availability

The original contributions presented in this study are included in the article/Supplementary Material. Further inquiries can be directed to the corresponding author.

## Supplementary Materials

The following supporting information can be downloaded at: https://www.mdpi.com/article/10.3390/foods15142532/s1, Figure S1: Effects of FA on the FTIR spectra of glutenin and gliadin; Figure S2: The deconvoluted amide I bands for glutenin in the dough system; Figure S3 The deconvoluted amide I bands for gliadin in the dough system; Figure S4: The deconvoluted amide I bands for glutenin in the simulated dough system, Figure S5: The deconvoluted amide I bands for gliadin in the simulated dough system; Figure S6 Effects of FA on the Raman spectra of glutenin and gliadin; Figure S7: Deconvoluted spectral region assigned to S-S bonds for glutenin in the dough system, Figure S8: Deconvoluted spectral region assigned to S-S bonds for gliadin in the dough system; Figure S9: Deconvoluted spectral region assigned to S-S bonds for glutenin in the simulated dough system; Figure S10: Deconvoluted spectral region assigned to S-S bonds for gliadin in the simulated dough system.
