# Supplementary material for: Unraveling the Molecular Interactions Between Ferulic Acid and Wheat Glutenin/Gliadin in Different Systems
Source: Foods. 2026 Jul 17;15(14):2532. doi: 10.3390/foods15142532 (PMC13408271; doi:10.3390/foods15142532)
Supplement: Supplementary file 1 [file foods-15-02532-s001.zip › foods-4359363-supplementary.pdf]

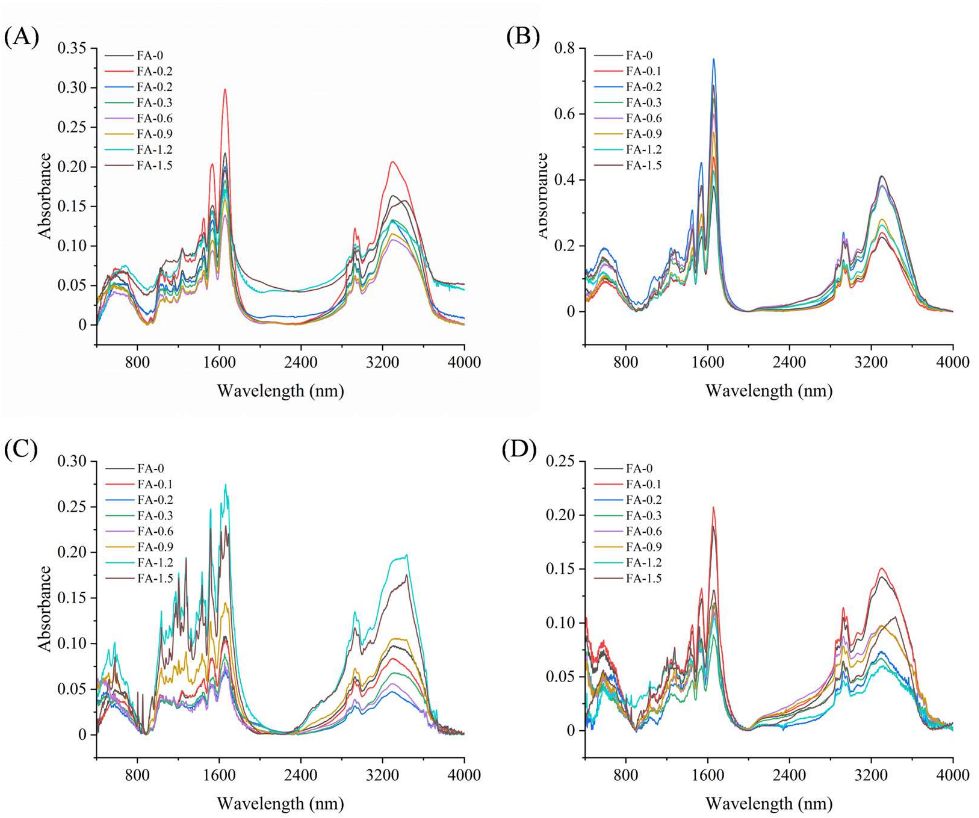

Figure S1 Effects of FA on the FTIR spectra of glutenin and gliadin:
(A) Glutenin in the dough system; (B) Gliadin in the dough system;
(C) Glutenin in the simulated dough system; (D) Gliadin in the simulated dough system.

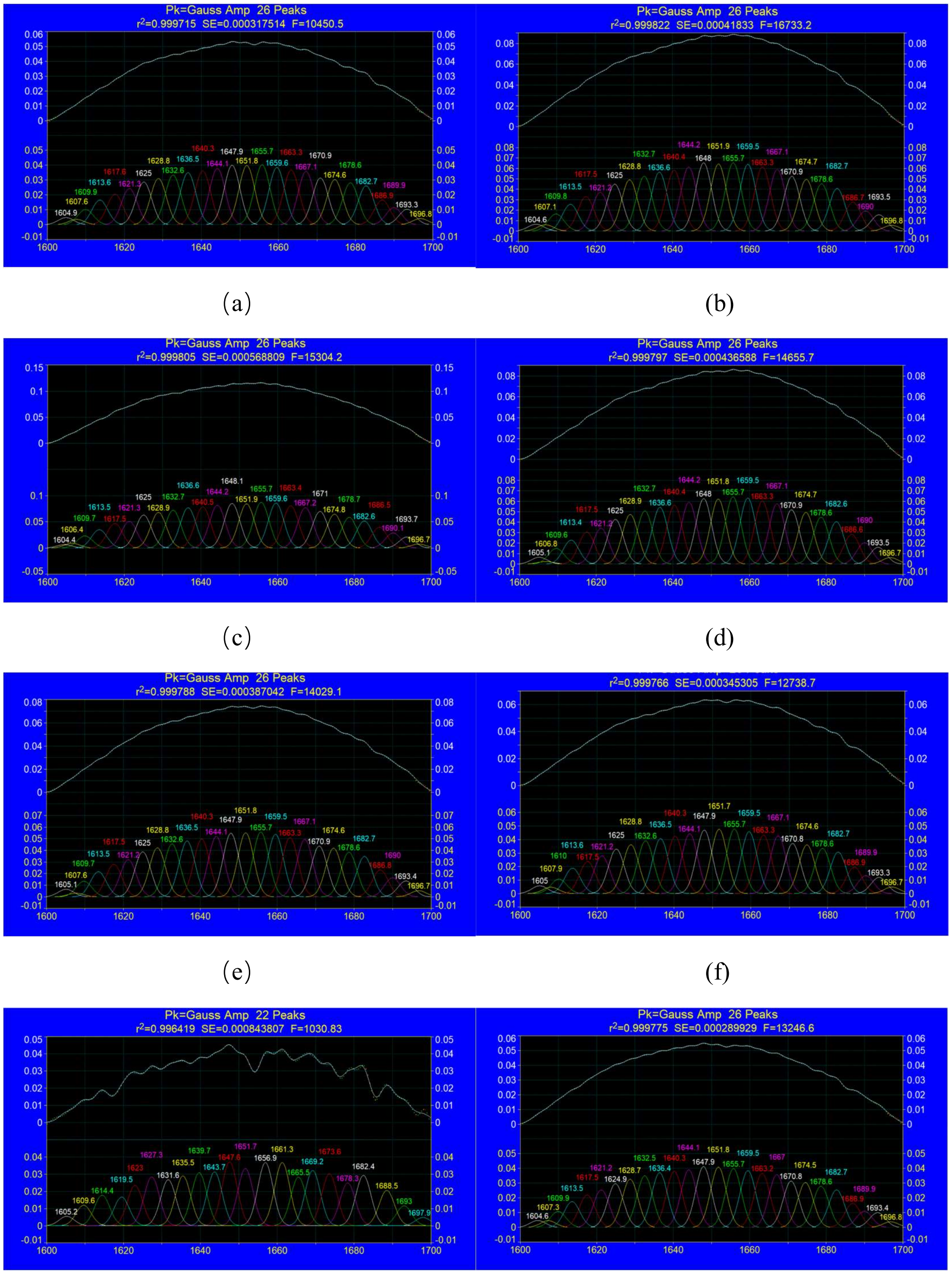

Figure S2 The deconvoluted amide I bands for glutenin in the dough system.
(a-h: addition of 0, 0.1, 0.2, 0.3, 0.6, 0.9, 1.2, and 1.5 g FA, respectively)

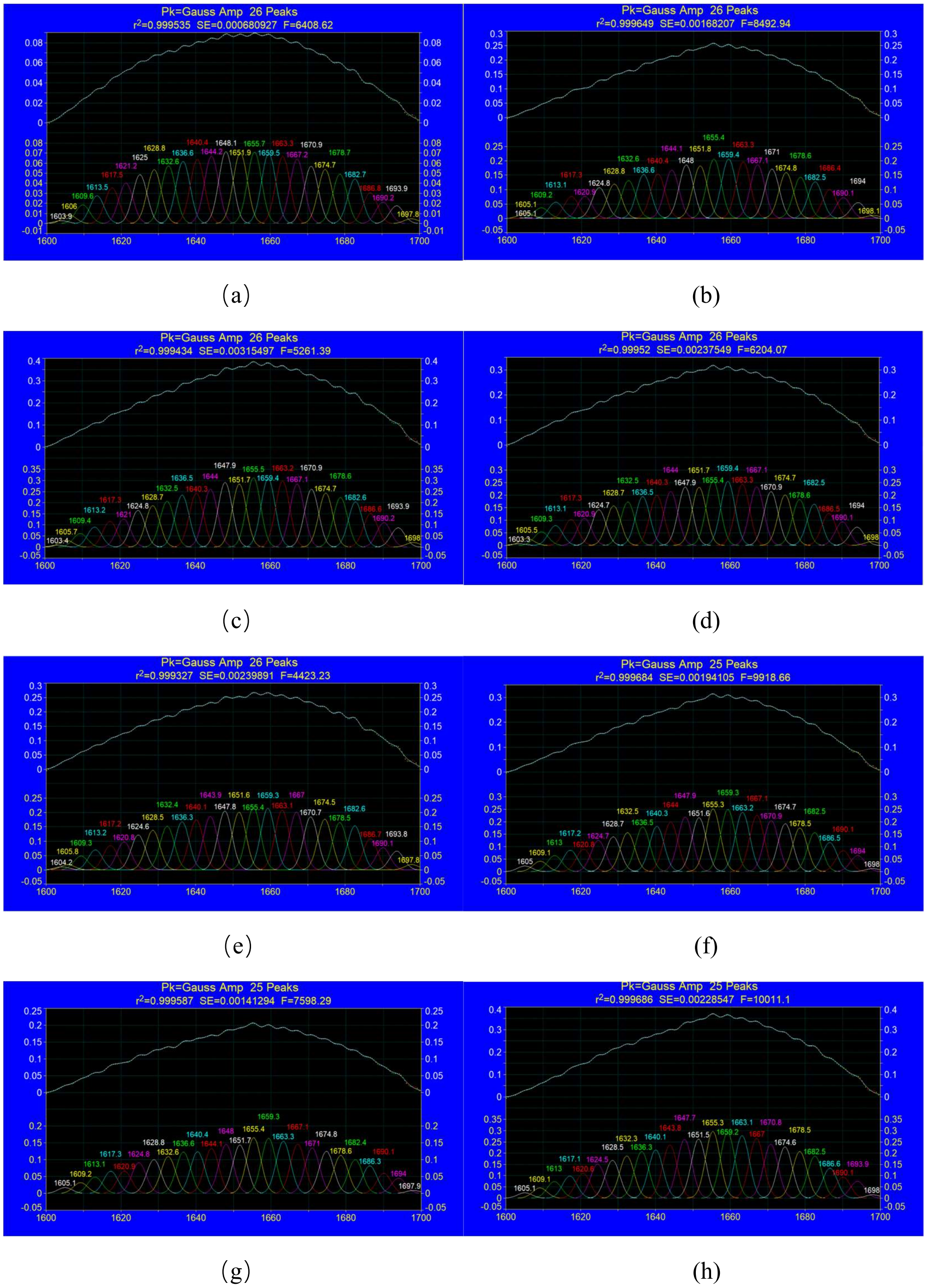

Figure S3 The deconvoluted amide I bands for gliadin in the dough system.
(a-h: addition of 0, 0.1, 0.2, 0.3, 0.6, 0.9, 1.2, and 1.5 g FA, respectively)

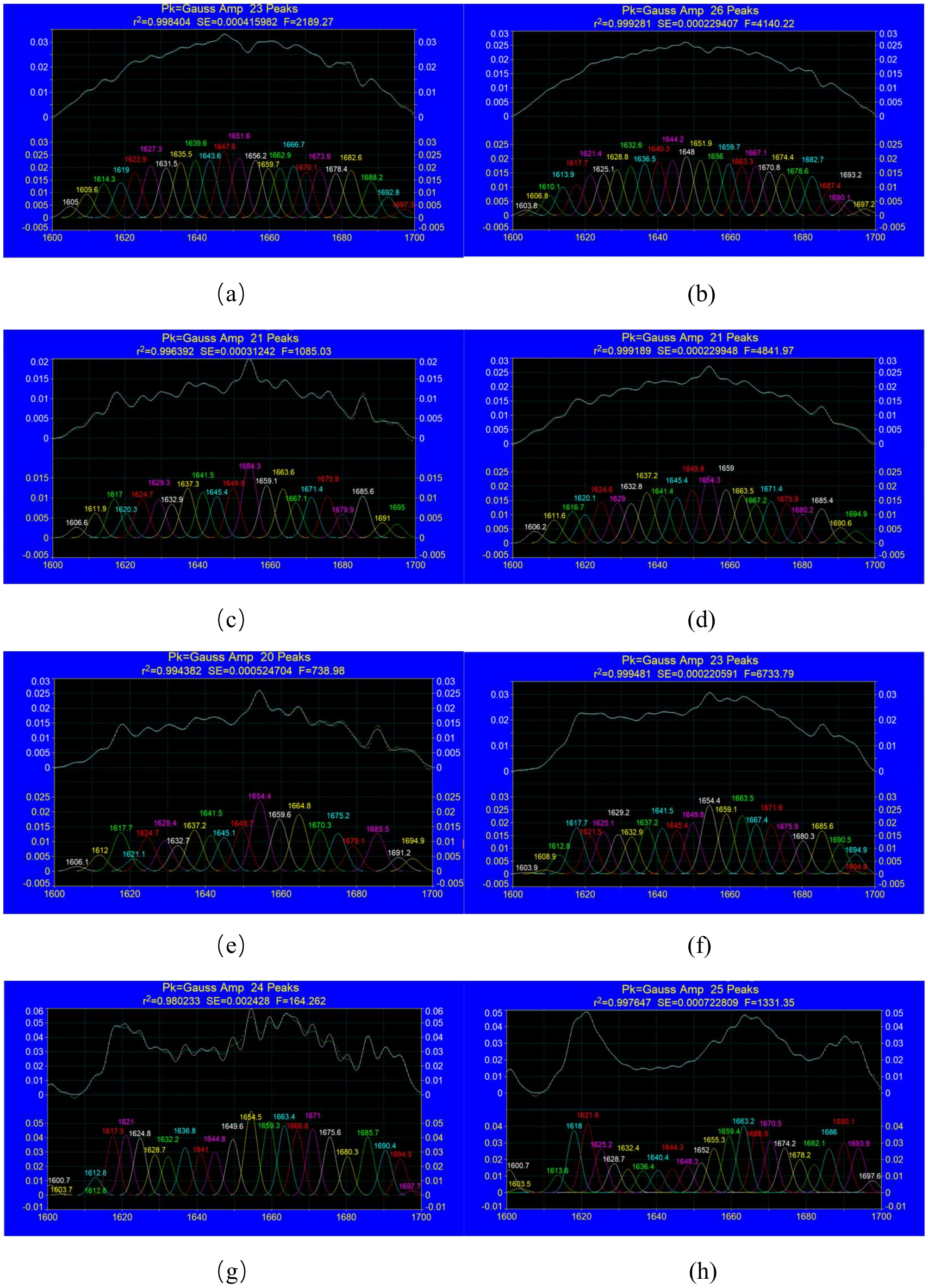

Figure S4 The deconvoluted amide I bands for glutenin in the simulated dough system.
(a-h: addition of 0, 0.1, 0.2, 0.3, 0.6, 0.9, 1.2, and 1.5 g FA, respectively)

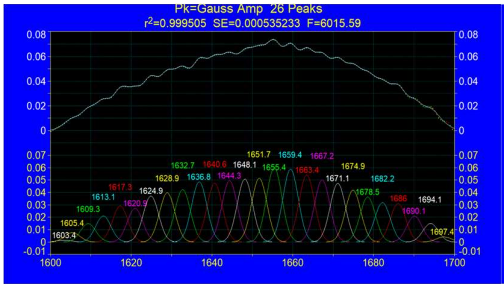

(a)

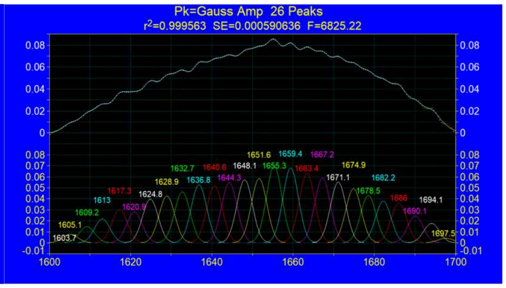

(b)

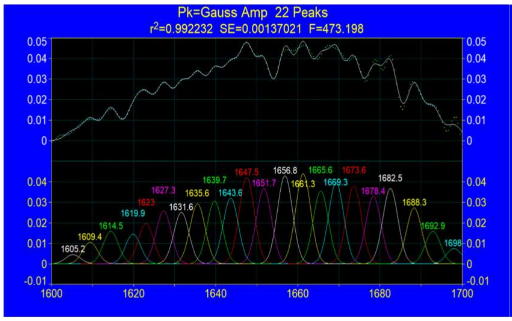

(c)

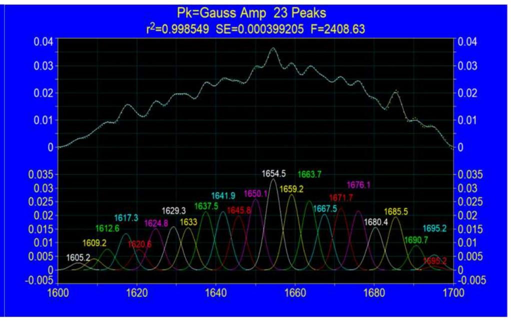

(d)

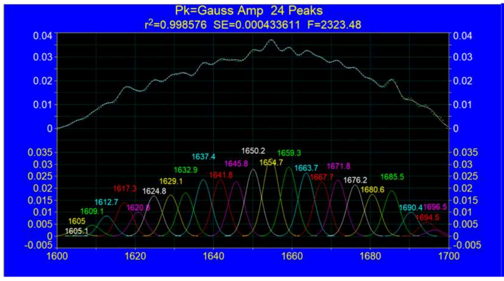

(e)

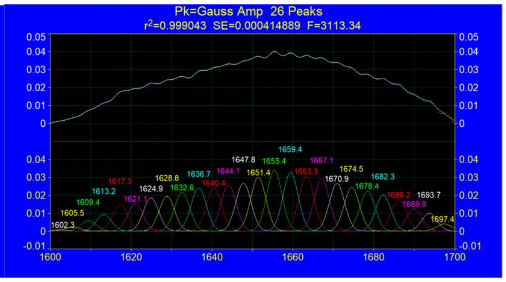

(f)

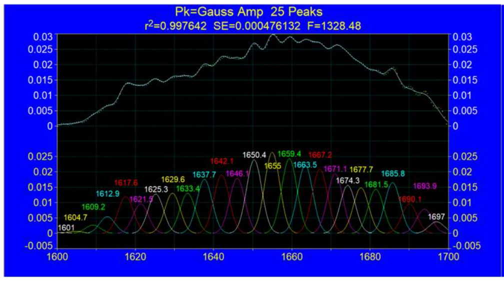

(g)

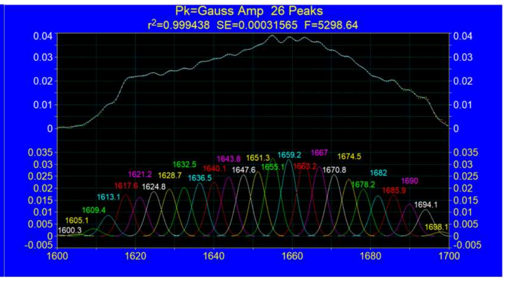

(h)

Figure S5 The deconvoluted amide I bands for gliadin in the simulated dough system.
(a-h: addition of 0, 0.1, 0.2, 0.3, 0.6, 0.9, 1.2, and 1.5 g FA, respectively)

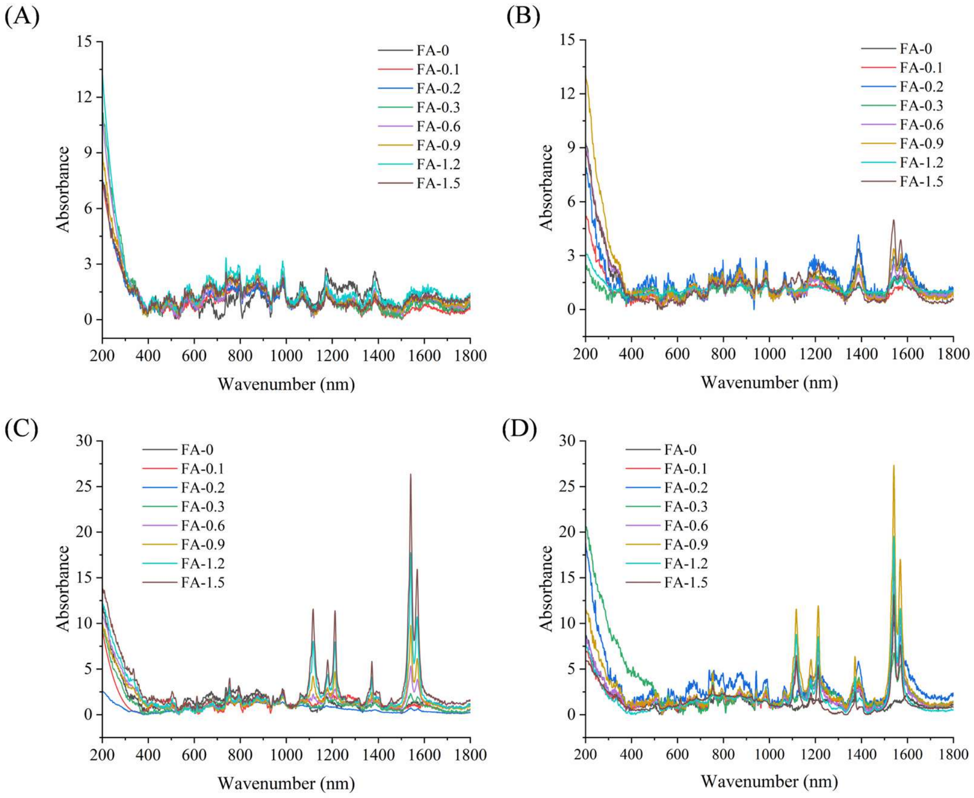

Figure S6 Effects of FA on the Raman spectra of glutenin and gliadin:

(A) Glutenin in the dough system; (B) Gliadin in the dough system;

(C) Glutenin in the simulated dough system; (D) Gliadin in the simulated dough system.

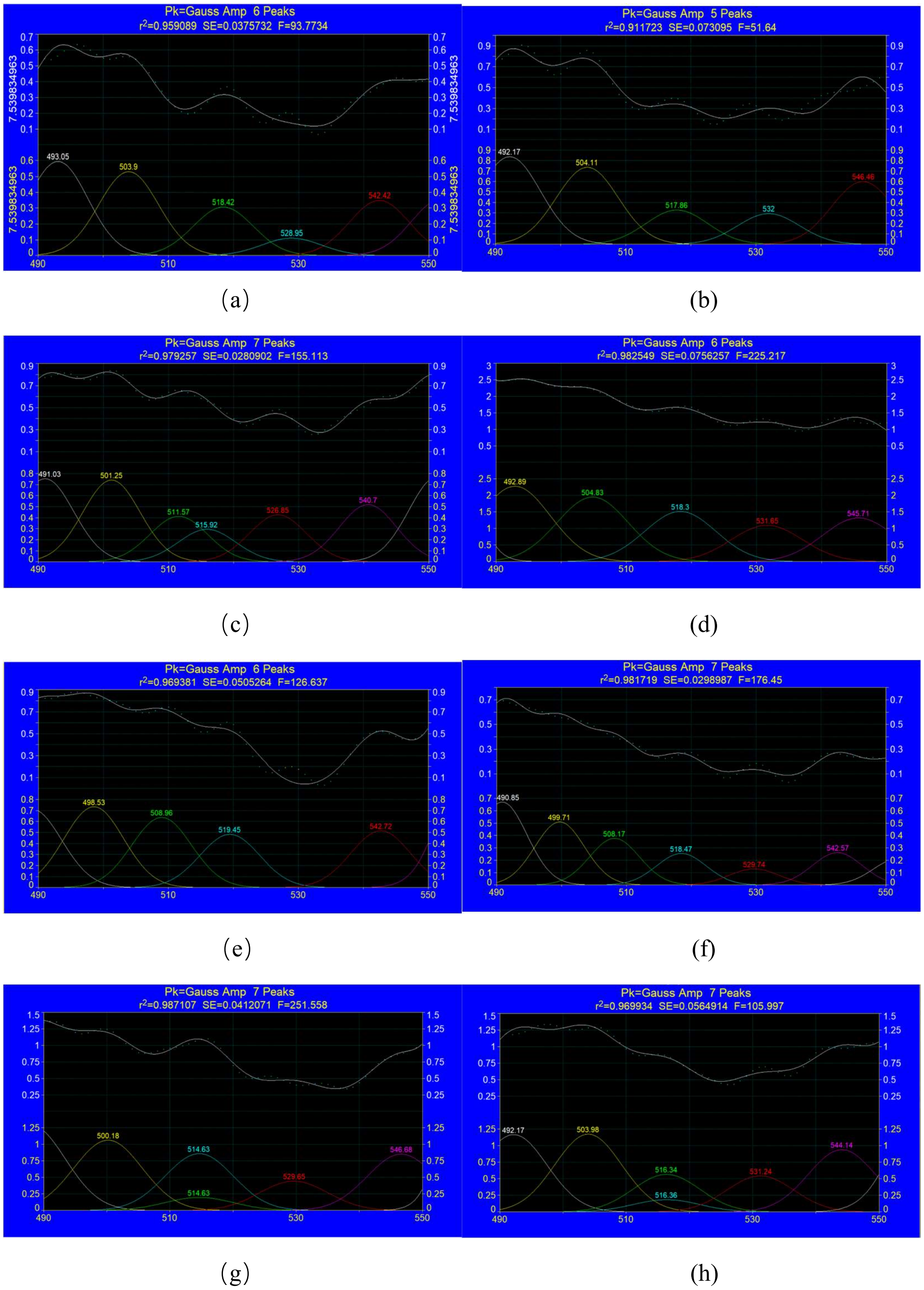

Figure S7 Deconvoluted spectral region assigned to S-S bonds for glutenin in the dough system. (a-h: addition of 0, 0.1, 0.2, 0.3, 0.6, 0.9, 1.2, and 1.5 g FA, respectively)

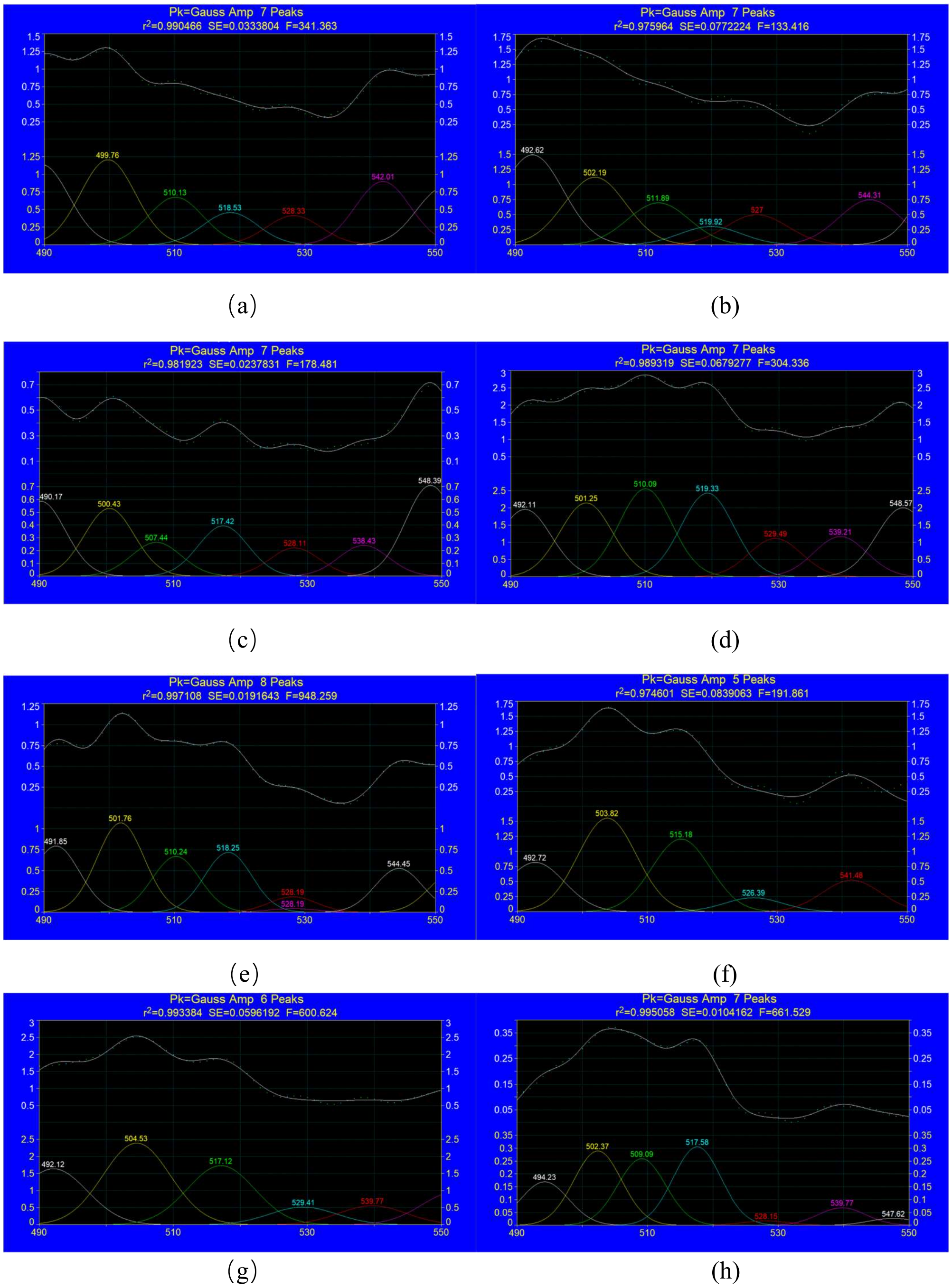

Figure S8 Deconvoluted spectral region assigned to S-S bonds for gliadin in the dough system. (a-h: addition of 0, 0.1, 0.2, 0.3, 0.6, 0.9, 1.2, and 1.5 g FA, respectively)

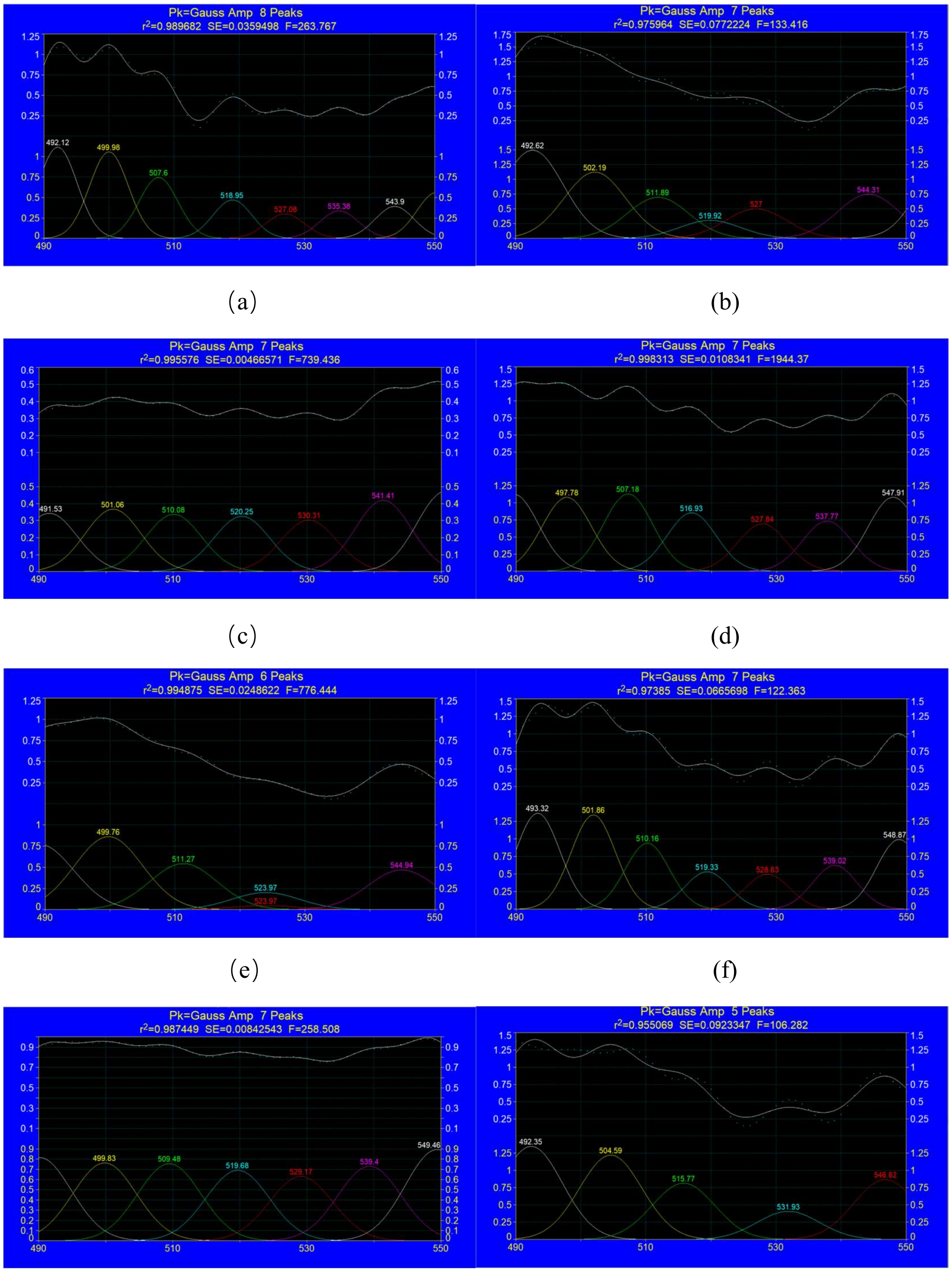

Figure S9 Deconvoluted spectral region assigned to S-S bonds for glutenin in the simulated dough system.

(a-h: addition of 0, 0.1, 0.2, 0.3, 0.6, 0.9, 1.2, and 1.5 g FA, respectively)

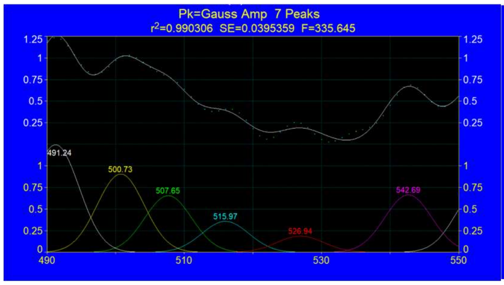

(a)

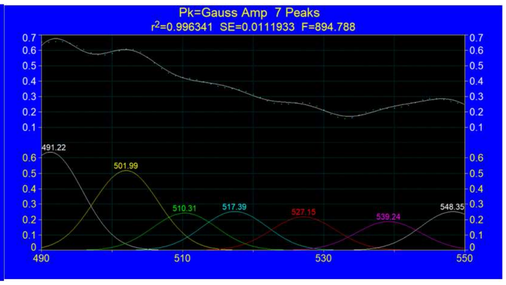

(b)

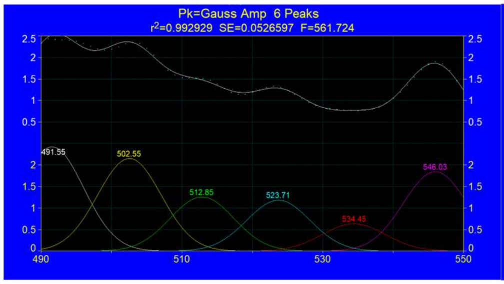

(c)

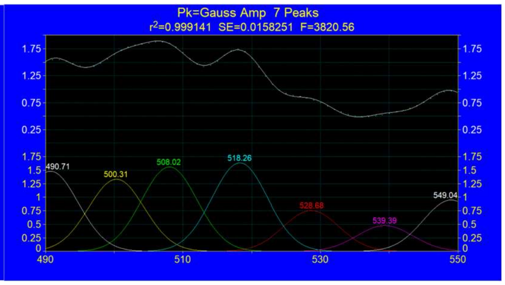

(d)

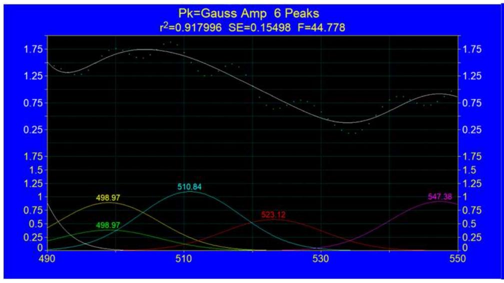

(e)

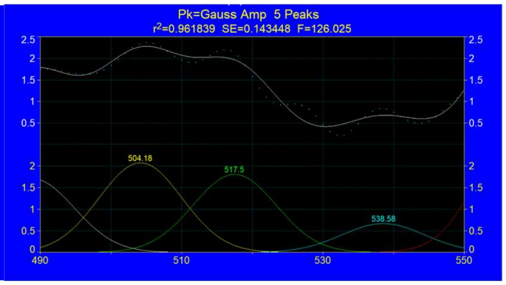

(f)

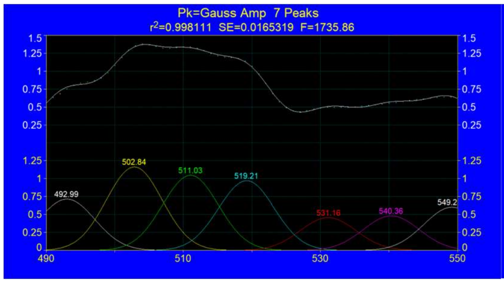

(g)

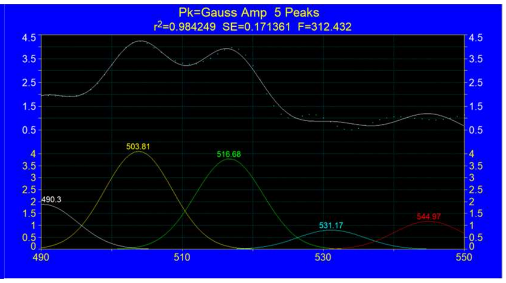

(h)

Figure S10 Deconvoluted spectral region assigned to S-S bonds for gliadin in the simulated dough system.

(a-h: addition of 0, 0.1, 0.2, 0.3, 0.6, 0.9, 1.2, and 1.5 g FA, respectively)
